# *VviERF6Ls*: an expanded clade in *Vitis* responds transcriptionally to abiotic and biotic stresses and berry development

**DOI:** 10.1186/s12864-020-06811-8

**Published:** 2020-07-09

**Authors:** Haley S. Toups, Noé Cochetel, Dennis Gray, Grant R. Cramer

**Affiliations:** 1 Department of Biochemistry and Molecular Biology, University of Nevada, Reno, NV 89557 USA; 2 Precision Bred LLC, 16676 Sparrow Hawk Lane, Sonora, CA 95370 USA

**Keywords:** ERF6L, Ethylene response factor, Motif, *Vitis vinifera*

## Abstract

**Background:**

*VviERF6Ls* are an uncharacterized gene clade in *Vitis* with only distant *Arabidopsis* orthologs. Preliminary data indicated these transcription factors may play a role in berry development and extreme abiotic stress responses. To better understand this highly duplicated, conserved clade, additional members of the clade were identified in four *Vitis* genotypes. A meta-data analysis was performed on publicly available microarray and RNA-Seq data (confirmed and expanded with RT-qPCR), and *Vitis VviERF6L1* overexpression lines were established and characterized with phenotyping and RNA-Seq.

**Results:**

A total of 18 PN40024 *VviERF6Ls* were identified; additional *VviERF6Ls* were identified in Cabernet Sauvignon, Chardonnay, and Carménère. The amino acid sequences of VviERF6Ls were found to be highly conserved. *VviERF6L* transcripts were detected in numerous plant organs and were differentially expressed in response to numerous abiotic stresses including water deficit, salinity, and cold as well as biotic stresses such as red blotch virus, *N. parvum*, and *E. necator*. *VviERF6Ls* were differentially expressed across stages of berry development, peaking in the pre-veraison/veraison stage and retaining conserved expression patterns across different vineyards, years, and *Vitis* cultivars. Co-expression network analysis identified a scarecrow-like transcription factor and a calmodulin-like gene with highly similar expression profiles to the *VviERF6L* clade. Overexpression of *VviERF6L1* in a Seyval Blanc background did not result in detectable morphological phenotypes. Genes differentially expressed in response to *VviERF6L1* overexpression were associated with abiotic and biotic stress responses.

**Conclusions:**

VviERF6Ls represent a large and distinct clade of ERF transcription factors in grapevine. The high conservation of protein sequence between these 18 transcription factors may indicate these genes originate from a duplication event in *Vitis*. Despite high sequence similarity and similar expression patterns, *VviERF6Ls* demonstrate unique levels of expression supported by similar but heterogeneous promoter sequences. *VviERF6L* gene expression differed between *Vitis* species, cultivars and organs including roots, leaves and berries. These genes respond to berry development and abiotic and biotic stresses. *VviERF6L1* overexpression in *Vitis vinifera* results in differential expression of genes related to phytohormone and immune system signaling. Further investigation of this interesting gene family is warranted.

## Background

Ethylene is a key phytohormone with roles in plant growth and development [1] as well as abiotic and biotic stress responses [2–4]. *Vitis vinifera* (grapevine) is a non-climacteric fruit that does not ripen with a respiratory burst of ethylene, instead maturing with increased abscisic acid (ABA) concentration. However, ethylene plays an important role in fruit development as berries transition into veraison, the beginning stage of color development and berry softening, initiating ethylene signaling and activating Ethylene Response Factors (ERFs) [5–7]. ERFs regulate gene expression of targets including transcription factors like *RESPONSIVE TO DEHYDRATION 29B* (*RD29B*), *LATE EMBRYOGENESIS 4–5* (*LEA4–5*), *HEAT SHOCK PROTEIN 101* (*HSP101*), and other *ERFs*, resulting in physiological responses and adaptations that allow a plant to better survive under specific environmental conditions like water deficit and high temperature [8]. These transcription factors act as major signaling hubs that integrate cross-talk between ethylene and other phytohormones to mediate gene expression [9–14]. ERFs belong to the APETLA2/ERF Family consisting of over 122 and 149 genes in Arabidopsis [15] and *Vitis* [4], respectively. This family is divided into 12 sub-families based on regulatory elements and DNA-binding domains.

Previously, a unique *Vitis* clade was identified in subfamily IX, consisting of 12 members with no Arabidopsis ortholog [16]. Sequence analysis revealed these genes most closely resembled *AtERF6.* This clade was named *ERF6-like* (*ERF6L*) after the closest Arabidopsis ortholog, and the genes were numbered from one through 12 based on chromosomal coordinates of the V1 structural annotation V2 assembly of PN40024 [16]. Affymetrix and NimbleGen grapevine microarrays with limited probe sets hybridizing to some of the *VviERF6L* genes revealed *VviERF6Ls* were differentially expressed in berry skins across berry ripening [16] and in leaves in response to severe leaf dehydration [17].

Recently, a new improved structural annotation (V3) of the PN40024 genome (V2 assembly) was released, providing additional gene loci [18]. In this report, the early observations of the *VviERF6L* clade are investigated further. These genes were analyzed using the new structural annotation of PN40024 [18]; to better understand the role of this clade in *Vitis*, gene expression patterns were queried in a meta-data analysis and novel experimental treatments were performed. *VviERF6L1* overexpression lines were established and phenotyped. Manual curation of the new structural annotations resulted in the discovery of additional *VviERF6Ls* not previously identified. *VviERF6L* expression was dependent on cultivar, species, organ, hormone and stress treatments.

## Results

### The *VviERF6L* clade was expanded to 18 members in the PN40024 reference genome

Novel *VviERF6Ls* genes were discovered by manually searching for conserved amino acid (AA) motifs in the newly annotated PN40024 genome. Along with the 12 previously identified *VviERF6L* genes [16], five additional genes were first identified (Table 1) from this manual curation. These additional genes were identified in unannotated sections of chromosome sequences by searching for specific AA motifs across the individual chromosomes using tools in ORCAE [19] where the reference grape genome sequence, PN40024, is stored. Structural models were confirmed in ORCAE using both mRNA [20] and expressed sequence tag (EST) data to confirm 5′ and 3′ ends of annotated sequences [21].

**Table 1:** PN40024 VviERF6L gene names and coordinates. Common gene names, V2 and V3 PN40024 loci annotation names, start and stop gene chromosome coordinates, coding strands, and total protein lengths in amino acid residues of the 18 identified *VviERF6Ls* in PN40024

PN40024 *VviERF6Ls*						
Gene Name	Loci name V3	Loci name V2	start	stop	strand	Length (AA)
VviERF6L1	Vitvi16g00350	VIT_16s0013g00900	6,283,579	6,284,605	+	278
VviERF6L2	Vitvi16g01429	VIT_16s0013g00950	6,323,868	6,324,845	+	278
VviERF6L3	Vitvi16g01438	VIT_16s0013g00970	6,340,458	6,341,282	+	275
VviERF6L4	Vitvi16g01430	VIT_16s0013g00980	6,353,674	6,354,603	+	276
VviERF6L5	Vitvi16g01424	VIT_16s0013g00990	6,374,230	6,375,289	+	278
VviERF6L6	Vitvi16g01432	VIT_16s0013g01000	6,390,945	6,391,977	–	269
VviERF6L7	Vitvi16g00362	VIT_16s0013g01050	6,495,312	6,496,325	+	278
VviERF6L8	Vitvi16g00363	VIT_16s0013g01060	6,518,283	6,519,283	+	278
VviERF6L9	Vitvi16g00370	VIT_16s0013g01070	6,550,999	6,552,087	+	276
VviERF6L10	Vitvi16g01437	VIT_16s0013g01090	6,626,070	6,627,309	–	265
VviERF6L11	Vitvi16g01434	VIT_16s0013g01110	6,659,232	6,660,318	–	279
VviERF6L12	Vitvi16g00380	VIT_16s0013g01120	6,692,405	6,693,620	+	243
VviERF6L13	Vitvi16g01423	NA	6,387,963	6,388,894	+	275
VviERF6L14	Vitvi16g00360	NA	6,460,898	6,461,806	+	191
VviERF6L15	Vitvi16g01442	NA	6,530,589	6,531,483	+	275
VviERF6L16	Vitvi16g01444	NA	6,621,652	6,622,826	–	283
VviERF6L17	Vitvi16g01443	NA	6,662,810	6,663,750	–	265
VviERF6L18	Vitvi16g01435	NA	6,667,987	6,669,132	–	279

**Fig. 1:**
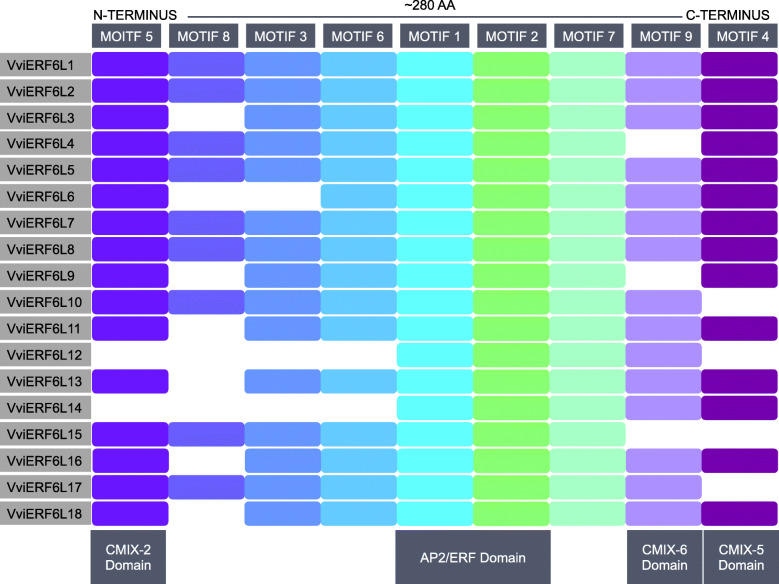
PN40024 VviERF6L protein motif relative presence and order. The relative order of the nine highly conserved amino acid motifs (top) in the 18 PN40024 VviERF6L proteins from N-terminus (left) to C-terminus (right) spanning in total an average of ~ 280 amino acid residues. White spaces indicate absence of a motif. Previously identified motifs are labeled (bottom). Exact motif coordinates are in Additional File 3

An additional VviERF6L was identified with an *in-silico* detection strategy. The manually curated VviERF6Ls were confirmed and substantiated as members of this clade from protein motifs identified in MEME [22, 23]. MEME revealed VviERF6L proteins consist of nine highly conserved AA motifs (Fig. 1 and Additional Files 1 and 2). The nine motifs are referred to in order of E-value with the lowest value motif corresponding to Motif 1 (Additional File 2). To identify additional novel *VviERF6Ls* that may have been overlooked with the manual annotation, an *in-silico* detection strategy was devised using the first (Motif 5) and last (Motif 4) spatial AA motifs to query the *Vitis* proteome. Genome coordinates that contained either the first, last, or both motifs were extracted corresponding to the potential proteins containing the motif(s) of interest. When only the first or the last motif was detected, the putative protein sequence was extended to 280 AA to obtain the potential full-length protein. This strategy confirmed the five novel *VviERF6L* genes from the manual curation and identified a sixth, increasing the members of the *VviERF6L* clade from 12 in the V1 annotation to 18 in the V2 annotation of PN40024 (Table 1).

The nine VviERF6L protein motifs were detected as being significantly present (*p* < 1.73 × 10^− 179^) and conserved (E < 8.8 × 10^− 14^) among the 18 VviERF6Ls (Additional File 2). These motifs had an average length of ~ 30 AA with the longest and shortest motifs (Motif 1 and Motif 9) having lengths of 50 and 7 AA, respectively (Additional File 2), resultant of the MEME settings used. Specific VviERF6L protein motif sequence and location per VviERF6L can be found in Additional File 3. Four protein motifs were identifiable and had previously been characterized as regulatory domains of other ERF Group IX TFs (Fig. 1) [15]. These protein motifs included the AP2/ERF domain (DNA-binding; Motifs 1 and 2), the CMIX-2 (N-terminal acidic transactivation; Motif 5) the CMIX-5 (MAP kinase phosphorylation site; Motif 4), and the CMIX-6 (MAP kinase phosphorylation site; Motif 9) domains (Fig. 1). Motifs 1, 2, and 7 were present in all 18 VviERF6Ls. Motifs 3, 4, 5, 6, and 9 were present in 77.8% of VviERF6L proteins, and Motif 8 was present in nine of the 18 VviERF6Ls (calculated from Additional File 3).

The VviERF6L AP2/ERF domain is homologous to that of Arabidopsis (At)ERF1 and 096. To identify VviERF6L sequence conservation with proteins in *Arabidopsis thaliana*, the nine motifs were queried in InterPro and the AP2/ERF domain was modeled in SWISS-model [24, 25]. The AP2/ERF domain (Motifs 1 and 2) of VviERF6L1, 2, 3, 4, 5, 6, 7, and 13 had the closest identity with that of AtERF1 with an average identity of 75.5% (Additional File 4). VviERF6L8, 9, 10, 11, 12, 15, 16, 17, and 18 had an average identity of 70.8% with the AtERF096 AP2/ERF domain, identified as the closest ortholog (Additional File 4).

VviERF6L12 and 14 appear to be truncated proteins. VviERF6L14 lacks the first 4 N-terminal motifs (Fig. 1), with no matching publicly available RNA-Seq or EST reads and insufficient sequence information in this region of the PN40024 genome in ORCAE. Besides *VviERF6L15*, *VviERF6L14* is the only other non-mono-exonal *VviERF6L*. The true start codon of *VviERF6L14* may exist in what is currently the un-sequenced region that is presently annotated as an intron and can be viewed in ORCAE [19]. Despite potential mis-annotation of *VviERF6L14* gene coordinates, promoter (see later) and protein motif analysis (Fig. 1, and Additional Files 1 and 2) validate this gene as a *VviERF6L*. VviERF6L12 appears to be a functional truncated protein (Fig. 1), supported by mRNA and EST read mapping across the length of the transcript in ORCAE. VviERF6L12 lacks the first 4 N-terminal motifs, which correspond to potential regulatory domains including the CMIX-2 domain (Fig. 1). VviERF6L12 is also missing Motif 4 corresponding to a CMIX-5 domain. VviERF6L3, 6, 9, 11, 13, 16, and 18 do not share consensus Motif 8 (Fig. 1). These proteins have higher amino acid variability in this region. VviERF6L10, 15, and 17 are also missing Motif 4 (Fig. 1).

**Fig. 2:**
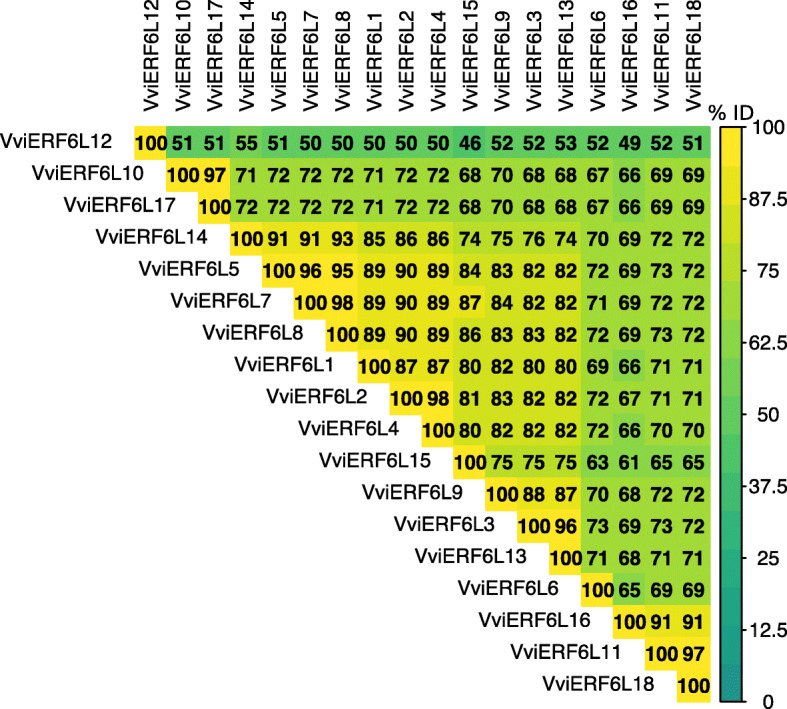
Percent identity of the 18 PN40024 VviERF6L proteins. Sequences were aligned with a MUSCLE multiple sequence alignment and compared to all other sequences with blue representing lower and yellow representing higher percent identity (% ID)

The 18 VviERF6L proteins are a conserved clade. A multiple sequence comparison by log-expectation (MUSCLE) multiple sequence alignment (MSA) was performed to better understand the diversity within the VviERF6L clade. Percent identity was extracted from a MUSCLE alignment (Fig. 2). The 18 VviERF6Ls share high sequence conservation (average of 73.8%), with VviERF6L12, one of the truncated VviERF6Ls, diverging the most with an average percent identity of 50.9% (calculated from Fig. 2).

The *VviERF6L* clade is expanded in *Vitis vinifera* relative to other plant species. The number of *ERF6L* paralogs were identified in the species that had the genes with the highest orthology to *VviERF6L1* from the Pan-taxonomic Compara Gene Tree in Gramene update 2018 containing 44 genomes [26] including the V1 annotation of PN40024 [20]. The number of potential *ERF6L* orthologs was quantified in carrot (*D. carota*), soybean (*G. max*), tomato (*S. lycopersicum*), and potato (*S. tuberosum*) (Additional File 5). *Vitis vinifera* had 4.5-fold more *ERF6L* paralogs than tomato and potato, 9-fold more than soybean, and 17 more potential *ERF6L* genes than carrot (Additional File 5).

### The *VviERF6L* clade is expanded across *Vitis* genotypes

Additional *VviERF6L*s were identified in the translated Cabernet Sauvignon (CS) genome [27] indicating the *VviERF6L* clade members vary with grape genotypes. The nine PN40024 VviERF6L protein motifs were utilized to detect *VviERF6Ls* in the proteome sequence of CS using TOMTOM [23]. Translated genes that contained at least three of the nine PN40024 VviERF6L protein motifs were extracted and analyzed with MEME as potential VviERF6Ls. These genes were used to identify CS specific VviERF6L protein motifs. TOMTOM used the CS cultivar specific VviERF6L protein motifs to identify additional potential CS VviERF6Ls that were missed using the nine PN40024 motifs (Additional Files 2 and 6, 7, 8, 9). Thirteen highly conserved (E < 1.3 × 10^− 2^) CS protein motifs (Additional File 2) were identified. The CS protein motifs were very similar to those of PN40024 (Additional Files 1, 7, 9 and 10). Homology between PN40024 and CS VviERF6L protein motifs was quantified with protein BLAST [28] (Additional File 10). CS Motifs 1–6 shared 100% identity with corresponding PN40024 motifs, and CS Motif 7 shared ~ 71% with PN40024 Motif 9 (Additional File 10). In total, 26 CS VviERF6Ls were identified (Additional Files 8, 9, and 11). Interestingly, unique *VviERF6L* sequences were identified in CS like VvCabSauv08_H0036F_008.ver1.0.g139880, which appears to be a novel *VviERF6L* not conserved in PN40024 (Additional Files 12 and 13). Lengths of CS VviERF6L proteins are in Additional File 11. CS VviERF6Ls (~ 300 AA residues) were on average 20 AA residues longer than PN40024 (~ 280 AA residues) (Table 1 and Additional File 11). CS lacked paralogs similar to PN40024 VviERF6L3, 8, 11, and 14 and had distinct VviERF6Ls (like VvCabSauv08_P0367F.ver1.0.g601540 and VvCabSauv08_H0036F_008.ver1.0.g139880), without a clearly distinguishable PN40024 equivalent. VvCabSauv08_H0036F_008.ver1.0.g139910 (596 AA residues) contained duplicated Motif 1–4, 5–9, and 11 (Additional Files 6 and 9). VvCabSauv08_H0036F_008.ver1.0.g139950 (839 AA residues) consisted of duplicate Motif 1, 2, 7, and 12 and triplicate Motif 3, 5, 6, and 11. These two genes were about two and three times the length of the average CS VviERF6L (~ 300 AA residues (calculated from Additional File 10)), respectively. VvCabSauv08_H0036F_008.ver1.0.g139990 was a severely truncated VviERF6L (106 AA residues), completely lacking any conserved N-terminal motif (Additional Files 6 and 9). VvCabSauv08_P0070F.ver1.0.g450750 was of comparable length (243 AA residues) to the average CS VviERF6L, but this gene had more variable sequence, containing only four of the thirteen conserved motifs (Additional Files 6 and 9).

Chardonnay (CH) [29] and Carménère (CA) [30] also have expanded *VviERF6L* clades with 15 and 14 *VviERF6Ls* respectively (Additional Files 12 and 13). VviERF6Ls from PN40024 and CS were queried in protein BLAST in genome sequences of CH and CA to identify VviERF6Ls in these genotypes. The genomes of CH and CA were not released at this time; only BLAST was publicly available. Additional novel *VviERF6Ls* may exist in these genotypes, which may be identified using the motif detection strategy described for PN40024 and CS when the genomes become fully available.

The PN40024, CS, CH, and CA VviERF6Ls were more similar across *Vitis vinifera* genotypes than to other VviERF proteins (Additional File 12). To distinguish relationships between the highly homologous members of the VviERF6L clade in the AP2/ERF subfamily IX [16], a maximum likelihood phylogenetic tree was generated from *Vitis vinifera* PN40024, CS, CH, and CA VviERF6L paralogs and PN40024 VviERF proteins (gene names and protein sequences available in Additional File 13). The tree was created using the Jones-Taylor-Thornton model with the Bootstrap method test in MEGA X [31]. Sequences were extracted from the PN40024 V2 assembly V3 structural annotation [18], CS genome [27], CH BLAST-tool [29], and the CA BLAST tool [30]. All predicted VviERF6L proteins grouped together from the four genotypes examined (Additional File 12). Vitvi05g01525, corresponding to a putative disease related PRF protein [21], clustered with CH and CA VviERF6Ls in the multi-gentoype VviERF6L clade. This gene is inadequately sequenced on ORCAE, having 4512 N’s in the coding sequence, and it is unclear if this gene is correctly positioned or annotated. The *VviERF6L* clade is distinct from other members of the AP2/ERF family (Additional File 12), and the VviERF6L protein sequences are highly conserved.

### *VviERF6L* promoter regions are distinct with several conserved motifs

**Fig. 3:**
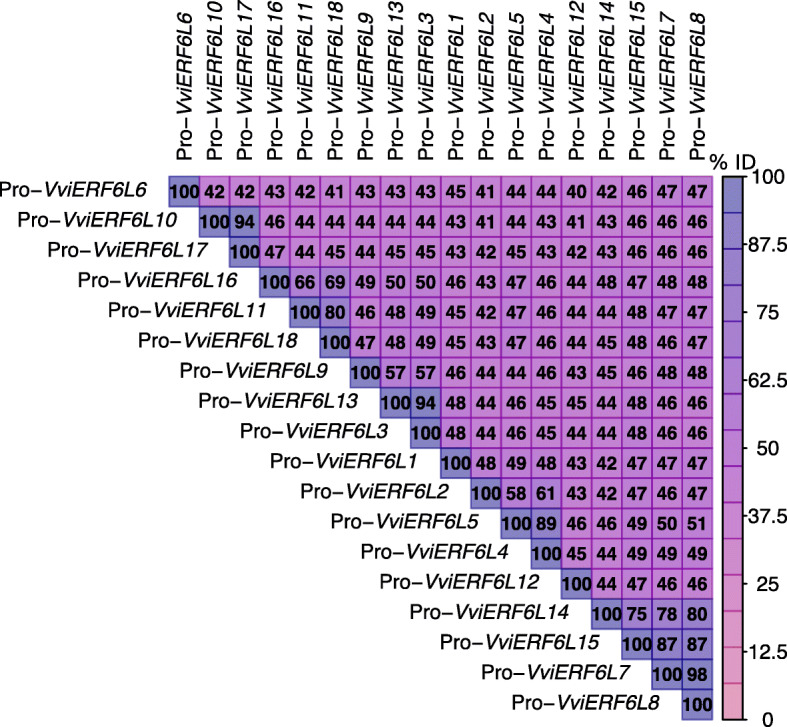
Percent identity of the 18 PN40024 *VviERF6L* putative promoter regions (− 3000 bp). Promoter (Pro-) sequences (− 3000 bp) were aligned with a MUSCLE multiple sequence alignment and compared to all other sequences with pink representing lower and purple representing higher percent identity (% ID)

The PN40224 *VviERF6Ls* have variable promoter regions with several conserved and repeated cis-acting elements. To gain insight into the transcriptional regulation of the highly conserved *VviERF6L* genes in the PN40024 genome, − 3000 bp upstream from the transcription start site (TSS) for the 18 PN40024 *VviERF6Ls* was analyzed with PLACE [32], and a multiple sequence alignment was performed to compare the putative promoter regions (Fig. 3). These regions showed greater diversity than VviERF6L protein sequences, averaging 48.7% relative to 81.05% identity (calculated from DNA sequences). PN40024 *VviERF6L* promoter region motif coordinates are in Additional File 14. A total of 200 known motifs were identified in the *VviERF6L* − 3000 bp putative promoter regions (calculated from Additional File 14). Of these cis-elements, 42 were present in all *VviERF6L* upstream regions (Additional File 15). The CAATBOX1 was the most represented motif across *VviERF6L* putative promoters, repeated 885 times, followed by DOFCOREZM (864 repetitions) and CACTFTPPCA1 (845 repetitions) (Additional File 15). These three motifs had an average of ~ 46 repeats per *VviERF6L* promoter. Numerous other cis-elements were repeated hundreds of times including ARR1AT and MYCCONSENSUSAT motifs.

Although the *VviERF6Ls* shared several highly repeated cis-regulatory elements, there were numerous differences across the *VviERF6L* promoter regions. The *VviERF6L12* promoter region contained the highest number of ROOTMOTIFTAPOX1 (82 repeats), with the closest *VviERF6L*, *VviERF6L14* having 41 repeats and the other *VviERF6Ls* having even fewer. The *VviERF6L12* promoter region also had the most duplication of TATABOX3, ACGTATERD1 (analogous with *VviERF6L1*), SEF1MOTIF, SEF4MOTIFGM7S, WBOXATNPR1, and LECPLEACS2 (Additional Files 15 and 16). Three unique motifs were detected in the promoter region of *VviERF6L12* that were not present in any other *VviERF6L* promoter: ABREZMRAB28, PALBOXLPC and UP1ATMASD (Additional File 14). Although the VviERF6L protein sequences are highly conserved, there is considerable variation in the *VviERF6L* promoter regions, indicating these genes are under unique transcriptional regulation.

### *VviERF6L* genes are expressed in many organs and tissues

Examining the grapevine gene atlas [33], *VviERF6Ls* were expressed in numerous grapevine organs, across developmental stages, and in tissues including berries, stamen, buds, tendrils, flowers, pollen, seeds, leaves, and roots (Additional File 17). *VviERF6L1*, *5*, and *12* were the most commonly expressed *VviERF6Ls* across various tissues. *VviERF6L6*, *10*, and *11* were less broadly expressed across tissues. *VviERF6L6* was only expressed in the rachis, carpel, petal, leaves, roots, and buds while *VviERF6L10* and *11* were expressed in these tissues as well as berry flesh. *VviERF6L8* was expressed in the same number of tissues as *VviERF6L2* and *3* and comparable to *VviERF6L4* and *7*. Breaking down the berry into pulp, seed, and skin *VviERF6Ls* generally had significantly higher expression in the skin at pre-veraison and seed at maturity when the berries are red, soft, and ready to harvest (RSH) (Additional File 18) [GSE49569] [34]. *VviERF6L12* was the only *VviERF6L* to increase in signal intensity in the pulp as berries developed, and this gene maintained the highest expression level in all berry tissues at all developmental stages.

### *VviERF6L* meta-data analysis parameters

*VviERF6L* expression was extensively examined across existing transcriptomic data in the literature. To better differentiate and understand the potential functional roles of *VviERF6L* genes in *Vitis*, *VviEFRF6L* gene expression was examined in a meta-data analysis of *VviERF6L* gene expression performed using 75 publicly available microarray and 24 RNA-Seq data series downloaded from NCBI Gene Expression Omnibus (GEO) [35] and Sequence Read Archive [36] data bases. The following data are examples of results found, but many other datasets demonstrate similar patterns. The example data series selected are simplified for visualization purposes. The data series (Additional File 19) investigated for *VviERF6L* gene expression met the following criteria: the experiment contained at least three individual biological or experimental replicates, *VviERF6L* gene expression was detectable, and at least one *VviERF6L* was identified as a differentially expressed gene (DEG) in the author’s original differential expression analysis (DEA). Results are discussed based on the author’s original DEA and statistical analysis unless otherwise specified.

Four and twelve probe sets were utilized on the grape Affymetrix and NimbleGen microarrays, respectively, with possible cross-hybridization occurring amongst the 18 PN40024 *VviERF6L*s [16]. Numerous occurrences of probe cross-hybridization for NimbleGen microarrays of the *VviERF6L* genes were previously determined [16] (Additional File 20), making it important to consider these results in terms of the *VviERF6L* clade response as opposed to individual gene responses. With the short-read length of the RNA-Seq data sets analyzed here and the high homology of the *VviERF6Ls*, it is unclear how distinguishable *VviERF6Ls* are individually in the RNA-Seq analysis.

Data series are referred to as in original publications. SRP117281, PRJNA516950, GSE67191, GSE62744, and GSE62745 were chosen as representative RNA-Seq data series of abiotic stress, berry development, and biotic stress to re-analyze with the V3 structural annotation of PN40024. The data series selected for re-analysis with the V3 structural annotation of PN40024 were used for weighted gene co-expression network analysis (WGCNA) to identify genes that share the same expression pattern as the *VviERF6Ls* under various stress conditions and developmental stages. All other data series demonstrated in the figures were graphed based on the original author’s transcript abundance quantification and statistics.

### *VviERF6L* genes are involved in multiple abiotic stress responses

#### VviERF6L genes respond to water deficit and salinity

**Fig. 4:**
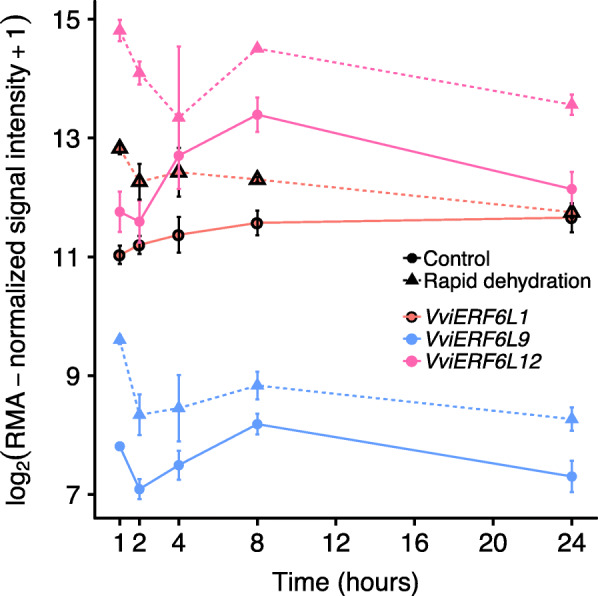
*VviERF6L* gene expression in response to rapid dehydration CS leaves. Log_2_(RMA-normalized signal intensity+ 1) gene expression of three *VviERF6Ls* in CS leaves treated with control (solid lines and circles), or detached and allowed to rapidly dehydrate under regulated conditions (dotted lines and triangles) for 1, 2, 4, 8, or 24 h. *VviERF6L1* in red with black outline, *VviERF6L9* in blue, and *VviERF6L12* in pink [GSE78920]; mean ± SE

*VviERF6Ls* were differentially expressed in response to numerous abiotic stresses including water deficit and salinity. *VviERF6Ls* were significantly differentially expressed in CS leaves exposed to rapid dehydration for 1 h (Fig. 4) [GSE78920] [17]. The *VviERF6Ls* shared the same general expression pattern in response to rapid dehydration with a significant increase in transcript abundance early in the experiment followed by decreased transcript abundance and plateauing thereafter. *VviERF6L12* and *9* demonstrated the highest and lowest levels of expression respectively. *VviERF6L1* was among the most responsive *VviERF6Ls*, increasing quickly to the severe stress within 1 h of treatment and decreasing at all time points after that to eventually be at the same level of expression as control plants after 24 h of treatment. *VviERF6L1* was chosen as a representative gene of the *VviERF6L* clade for subsequent RT-qPCR and overexpression experiments. RT-qPCR was performed for *VviERF6L1* on CS leaves treated with 10 μM Protone (s-ABA). *VviERF6L1* transcript abundance in CS leaves did not respond to ABA treatment (Additional File 21), indicating the water deficit response may follow an ABA independent pathway.

**Fig. 5:**
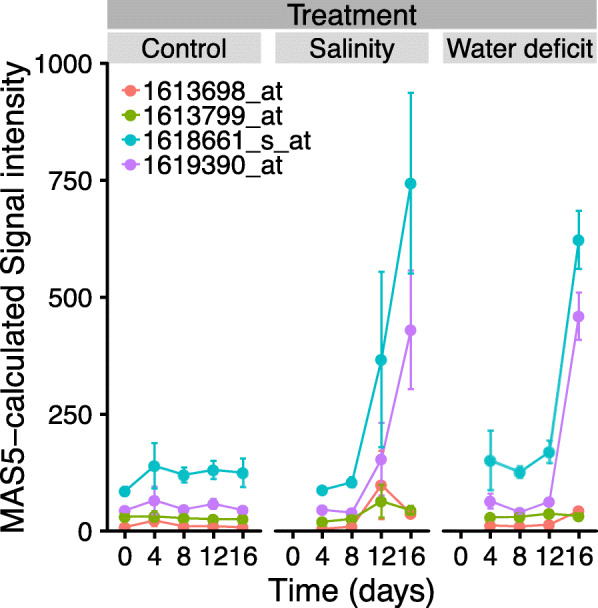
*VviERF6L* gene expression in response to salinity and water deficit in CS shoot tips. MAS5-calculaed signal intensity of four *VviERF6L* probes in which CS vine were treated with a control nutrient solution, a progressive ramp of NaCl + CaCl_2_, or a natural dry down for 16 days [GSE31677]; mean ± SE

CS shoot tips exposed to a severe 16-day salt and water deficit treatment also show significantly increased *VviERF6L* transcript abundance (Fig. 5) [37]. Probe sets 1618661_s_at (*VviERF6L12*) and 1619390_at (*VviERF6L11*) were highly induced by extreme water deficit and salt stress, but 1613698_at (cross-hybridizes to *VviERF6L2 and VviERF4*) and 1613799_at (*VviERF6L3*) were not induced. The accumulation of *VviERF6L* transcripts on day 16, the point at which both abiotic stresses were most severe indicate some *VviERF6Ls* may play a role in extreme salt and water deficit responses in grapevine.

**Fig. 6:**
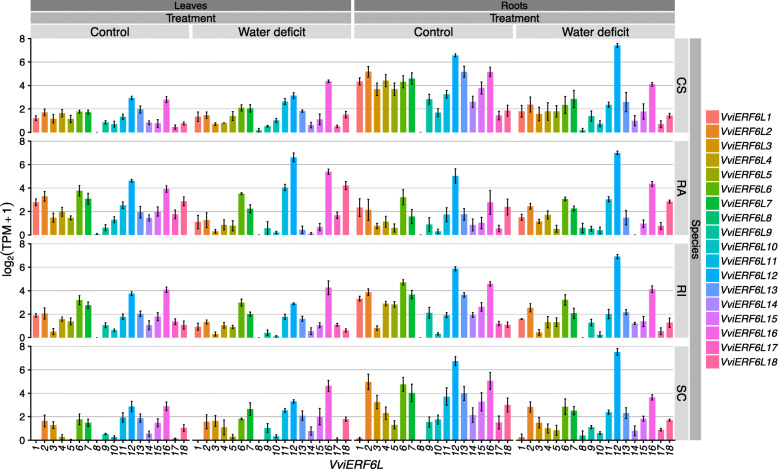
*VviERF6L* gene expression in response to 2 weeks of water deficit in leaves and roots of four *Vitis* species. Log_2_(Transcripts per million (TPM) + 1) of 18 *VviERF6Ls* treated with well-watered control and water deficit for 2 weeks in leaves (left) and roots (right) in CS, RA, RI, and SC [PRJNA516950]; mean ± SE

*VviERF6L* differential expression in response to water deficit is supported by recent more comprehensive RNA-Seq data in which four *Vitis* species (*Vitis vinifera* cv. CS, *Vitis champinii* cv. Ramsey (RA), *Vitis riparia* cv. Riparia Gloire (RI), and *Vitis vinifera x Vitis girdiana* cv. SC2 (SC)) were treated with well-watered and moderate water deficit (WD) conditions in the form of a natural dry-down for 2 weeks [38]. The grapevines demonstrate significantly differential *VviERF6L* expression patterns within each species (Fig. 6). For example, *VviERF6L1* is not expressed in SC, but it is expressed in the three other species. Within each Species x Organ x Treatment group, the 18 *VviERF6Ls* follow similar expression patterns to each other (Fig. 6). *VviERF6Ls* were differentially expressed in leaves and roots in response to the WD (Fig. 6). *VviERF6Ls* were significantly more highly expressed in roots than in leaves. Consistently, the *VviERF6Ls* have higher expression in roots than leaves under both Control and WD conditions (13 average TPM (standard error of the mean (SEM) = ± 1.5) vs 4.6 average TPM (SEM = ± 0.51) for roots and leaves, respectively). As a general trend *VviERF6L* transcripts were decreased in response to WD (e.g. *VviERF6L1*). The majority of *VviERF6Ls* have relatively low expression levels apart from *VviERF6L12* that demonstrated a significantly higher level of expression (Z-score for two population proportions *p* < 0.00001). *VviERF6L8* was consistently the lowest expressed *VviERF6L* across organs and treatments. Interestingly, leaves from RA (a drought tolerant rootstock originating from Texas, USA) had a significantly higher accumulation of *VviERF6L12* transcripts in week 2 WD relative RA Control leaves as well as compared to 2 WD treatment leaves of the other three species. The other species, which are more drought sensitive, did not exhibit an increase or as high of an increase in *VviERF6L12 *transcripts in the leaves in response to 2 weeks of WD.

Amongst the *VviERF6L* clade, DEA showed *VviERF6L1* and 18 were the most common DEGs in DEA contrasts of interest. DEA was performed for each genotype for WD vs. Control and for each WD treated species to the others for weeks one and two for roots and leaves individually. A list of DEA contrasts of interest for this data set are located in Additional File 22. The frequency at which each *VviERF6L* was a DEG in the DEA contrasts of interest was quantified (Additional File 23). *VviERF6L1* was identified as the most responsive *VviERF6L*, being a DEG in 14 DEA contrasts of interest followed by *VviERF6L18* (a DEG in 10 DEA contrasts of interest); however, both genes were expressed at relatively low levels (Fig. 6). The other *VviERF6Ls* varied in DEG frequency in the DEA contrasts of interest (Additional File 23). *VviERF6L7* and *8* were not DEGs in any contrast of interest. The range of frequencies each *VviERF6L* was a DEG in the DEA contrasts of interest was consistent with the results of the promoter analysis, indicating that while these genes are highly conserved, they are under distinct regulation.

### *VviERF6L* genes respond to chilling and cold

**Fig. 7:**
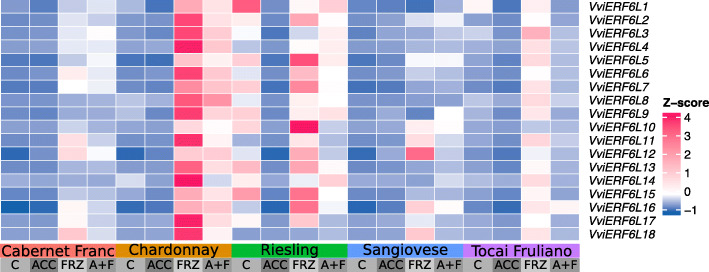
Heatmap representation of the gene expression of *VviERF6Ls* in response to cold. Average TPM of 18 *VviERF6Ls* were log_2_ transformed and represented as Z-scores (calculated per gene) with pink representing higher and blue representing lower values from leaves of whole vines treated with chilling acclimation (ACC), acclimation followed by freezing (A + F), control (C), and freezing (FRZ) from Cabernet Franc (pink), Chardonnay (orange), Riesling (green), Sangiovese (blue), and Tocai Friulano (purple) [SRP117281]

*VviERF6Ls* were differentially expressed in leaves in response to chilling, cold, and freezing in the meta-data analysis. Many of the *VviERF6Ls* responded with analogous expression patterns. Recent RNA-Seq data in which five *Vitis vinifera* cultivars (Cabernet Franc, Chardonnay, Riesling, Sangiovese, and Tocai Friulano) were treated with chilling (ACC), freezing (FRZ) or a chilling acclimation followed by the freeze treatment (A + F) demonstrate significant *VviERF6L* differential expression (Fig. 7) [39]. As with WD response, various *V. vinifera* cultivars demonstrated unique *VviERF6L* expression in leaves in response to cold treatments. For example, comparing freezing vs. control DEA across cultivars, Chardonnay had the highest significant increase in *VviERF6L1* transcript abundance while Tocai Friulano and Sangiovese did not demonstrate *ERF6L1* differential expression (Fig. 7 and DEA from original publication [39]). All cultivars had a decrease in transcript abundance of *VviERF6L1* in the chilling vs. control treatment comparison (Fig. 7). The transcript abundance of *VviERF6L11 and VviERF6L12* was increased in all genotypes in response to the freezing treatment (Fig. 7). The results in Fig. 7 are supported by microarray data in which *VviERF6Ls* were differentially expressed in CS shoot tips exposed to a chilling treatment for 0, 4, and 8 h (Additional File 24) [40]. To confirm these results, RT-qPCR was performed on *VviERF6L1* for RA, RI, CS, and SC whole canopy and single leaves treated with 4 °C chilling for 0–2 h. In contrast to the previous freezing and chilling treatments, these chilling experiments did not result in a significant difference of *VviERF6L1* transcript abundance relative to control; there was however, a significant increase in *CBF1* transcript abundance used as a positive control in chilled samples (Additional File 25). It is possible *VviERF6L1* was not the most responsive *VviERF6L* in these species under these conditions. Together these examples from the meta-data analysis reveal *VviERF6Ls* are differentially expressed with complex responses to temperature reduction in cultivar-, temperature- and time-dependent manners and may play a role in cold response in grapevine.

### *VviERF6L* genes respond to light intensity

**Fig. 8:**
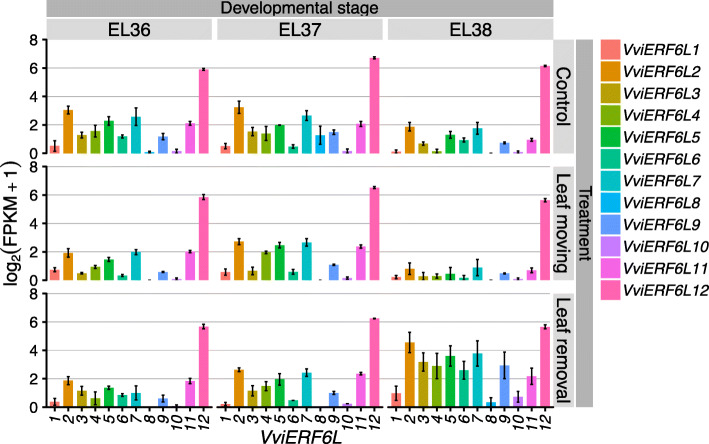
*VviERF6L* gene expression in response to light exposure. Log_2_(FPKM+ 1) gene expression of 12 *VviERF6Ls* from berry pericarp at three stages of ripening (EL36–38) under control, leaf movement, and leaf removal light exposure treatments [GSE121146]; mean ± SE

*VviERF6Ls* were significantly differentially expressed in response to increased light exposure. CS berries exposed to varying light intensity through leaf removal or leaf movement at veraison demonstrated reduced *VviERF6L12* transcript abundance in de-seeded berries (pulp and skin only) at late veraison and harvest. The majority of the other *VviERF6Ls* (all with lower transcript abundance than *VviERF6L12*) had enhanced transcript accumulation at harvest (particularly with leaf removal) relative to control conditions in which no leaves were (re) moved [41] (Fig. 8) [GSE121146]. In this experiment, leaves were cut off vines for the leaf removal treatment and physically bound in place for the leaf moving treatment. These actions may have elicited a wounding response. However, as the berries remained intact on the vine and only the leaves were removed, it is likely that the increased *VviERF6L* transcript abundance in the berries is associated with increased light exposure and not a wounding response. The accumulation of *VviERF6L* transcripts with enhanced light exposure at harvest in combination with the abundance of *VviERF6L* promoter motifs associated with light response indicates *VviERF6Ls* may play a role in grapevine response to light intensity. Supportive of these data and *VviERF6L* light response, *VviERF6Ls* were also differentially expressed in berries grown under a double cropping system with summer and winter harvests [GSE103226] [42]. In the summer, CS berries grown in this system had the highest level of *VviERF6L* expression at the end of veraison (EL36), while there was a distinct depression in *VviERF6L* transcript level for winter berries (Additional File 26). These data sets support the hypothesis that *VviERF6Ls* have a cultivar-specific response to abiotic stress and may play a role in response to light intensity.

### *VviERF6L *genes are involved in various biotic stress responses

**Fig. 9:**
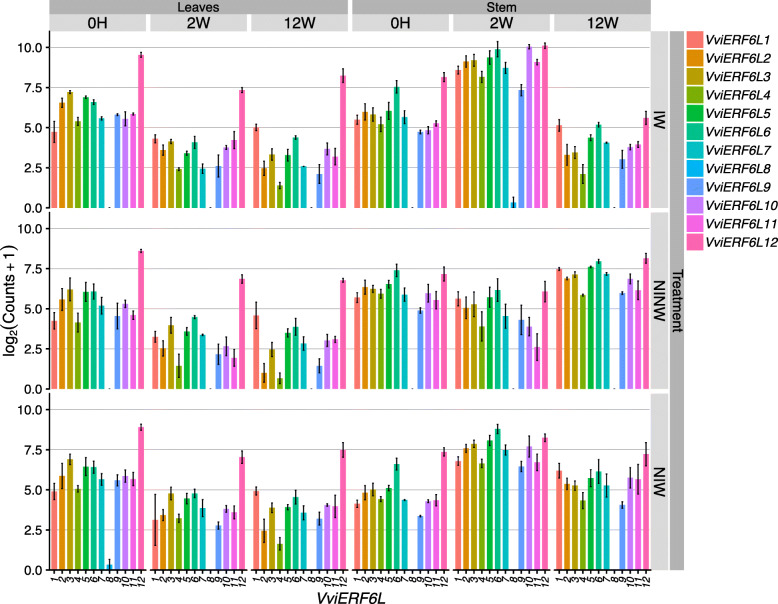
*VviERF6L* gene expression in response to *Neofusicoccum parvum* infection. Log_2_(Counts+ 1) gene expression of 12 *VviERF6Ls* from leaves and stems treated with control, non-infected – non-wounded (NINW), *Neofusicoccum parvum* infected and wounded (IW), and non-infected – wounded (NIW) after 0 h, and 2 and 12 weeks [GSE97900]; mean ± SE

*VviERF6Ls* are differentially expressed in response to *Neofusicoccum parvum*. CS plants inoculated with *N. parvum* had significantly enhanced *VviERF6L* transcript accumulation in woody stems 2 weeks after treatment (Fig. 9) [GSE97900] [43]. Interestingly, *VviERF6Ls* also responded to the wounding aspect of this treatment, which consisted of taking a power drill to the woody stem. The wounding response remained significant for the majority of *VviERF6Ls* up to 2 weeks after the treatment (Fig. 9). *VviERF6L12* and *8* consistently demonstrated the highest and lowest expression levels, respectively (Fig. 9).

In general, *VviERF6L* expression levels significantly increased in response to *E. necator* inoculation in leaves of *V. vinifera* cv. Carignane and six partially resistant Asian accessions (DVIT3351.27 (DVIT3351), Hussiene, Karadzhandal, Khalchii, O34–16, Sochal, and Vavilov) [GSE67191] [44]. The cultivars showed similar expression patterns with differences in expression levels of the *VviERF6Ls* (Additional File 27). *VviERF6L12* and *16* generally had the highest expression in response to the powdery mildew inoculation (Additional File 27). *VviERF6L8* generally had low, but detectable expression with the exception of DVIT3351 in which *VviERF6L8* had higher expression levels, similar to those of *VviERF6L3–7*, and Vavilov that did not demonstrate any *VviERF6L8* expression at either time point or treatment. It is possible *VviERF6Ls* play diverging roles in response to various pathogens.

**Fig. 10:**
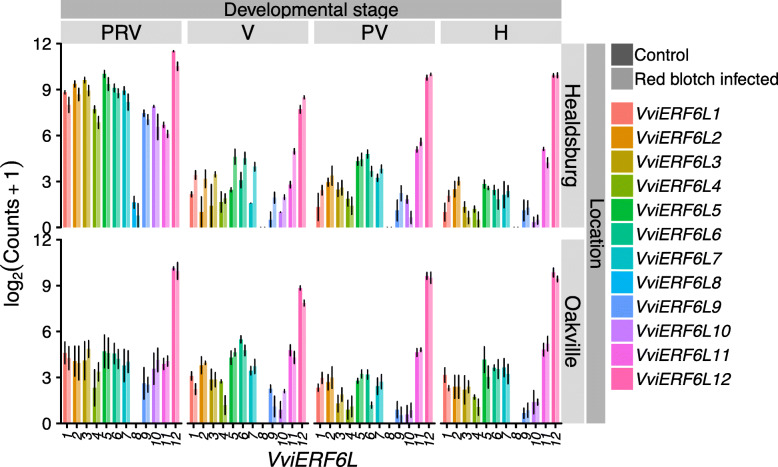
*VviERF6L* gene expression in response to red blotch-associated virus infection. Log_2_(Counts+ 1) gene expression of 12 *VviERF6Ls* from whole Zinfandel berries across four stages of berry development: pre-veraison (PRV), veraison (V), post-veraison (PV), and harvest (H) treated with control mock inoculation (dark) or red blotch associated virus infection (light) from two vineyards (Healdsburg and Oakville) [GSE85812]; mean ± SE

Whole Zinfandel berries had high levels of *VviERF6L12* counts across berry development in both control and red blotch-associated virus treatment in two separate vineyards (Fig. 10) [GSE85812] [45]. In control berries, the expression of the *VviERF6L* clade was highest in the pre-veraison (PRV) stage with subsequent decline in transcript abundance as the berry maturity stage increased. *VviERF6L8* expression was only detectable in one vineyard at pre-veraison; in all other cases it does not appear to be expressed (Fig. 10). *VviERF6L* expression at pre-veraison significantly decreased in response to red blotch-associated virus in at least one vineyard (Fig. 10) [45]. *VviERF6Ls* showed variable expression across the vineyards in response to red blotch-associate virus particularly at pre-veraison. *VviERF6L5*, *7*, and *10* had increased counts in infected samples at veraison (Fig. 10). At post-veraison, *VviERF6L1*, *5*, and *11* had higher expression in red blotch-associated virus samples in both vineyards, and at harvest, *VviERF6L3* had lower counts in infected berry tissue (Fig. 10). While various *VviERF6Ls* were significantly differentially expressed in response to these pathogens (Figs. 9, 10 and Additional File 27), the pattern and degree of expression across genotypes was not consistent.

### *VviERF6L* genes are involved in berry development

**Fig. 11:**
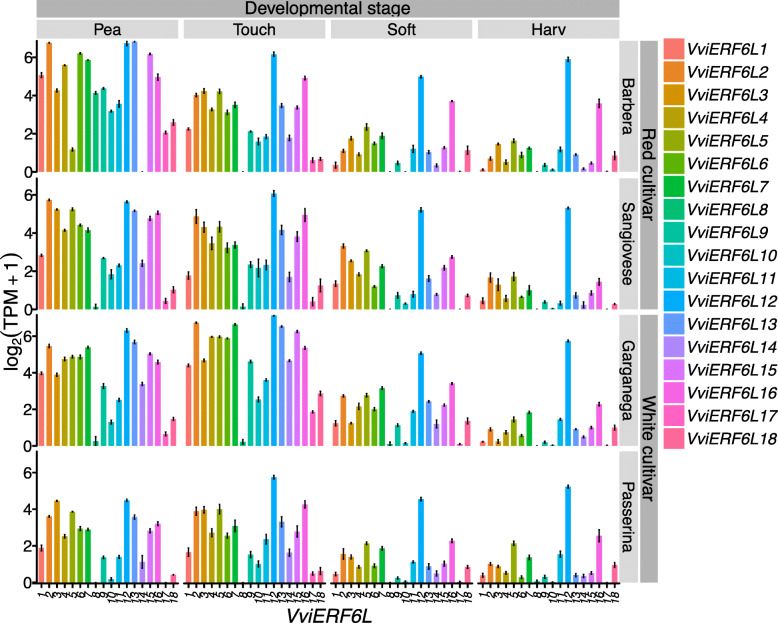
*VviERF6L* gene expression in response to berry development in red and white berries. Log_2_(Transcripts per million (TPM) + 1) of 18 *VviERF6Ls* from red cultivar berries (Barbera and Sangiovese) and white berries (Garganega and Passerina) at the pea-size (Pea), touching (Touch), softening (Soft), and harvest (Harv) stages of berry development [GSE62744 and GSE62745]; mean ± SE

Unlike abiotic and biotic stress responses, *VviERF6L* gene expression patterns and levels show general conservation across cultivars with *VviERF6L12* consistently having the highest transcript abundance in red and white berries [46]. One study examining red and white berry development over four developmental stages (pea sized (Pea), touching (Touch), softening (Soft), and harvest (Harv)) showed differential expression of *VviERF6Ls* across berry ripening, but at a low expression level (Fig. 11). *VviERF6Ls* had the highest number of *VviERF6L* transcripts at the pea-sized and touching stages of berry development. *VviERF6L* transcript abundance decreased as berries softened and was even lower at harvest (Fig. 11). *VviERF6L2*, *12*, and *13* were among the highest expressed *VviERF6Ls* in white berries with the addition of *VviERF6L5*, *15*, and *16* for red cultivars in the early stages of berry development. In the later stages of berry development, *VviERF6L12* clearly had the highest transcript abundance (Fig. 11). From pea-sized to touching berries, *VviERF6L8* was expressed in white berries (except Passerina) to a comparable level with other *VviERF6Ls*, including *VviERF6L7* and *9*. At all other developmental stages, *VviERF6L8* was negligibly expressed. In red berries, *VviERF6L8* was only expressed in Barbera in pea-sized berries. *VviERF6L* expression across berry development is also consistent across vineyards and years [GSE97578] [47] [GSE41633] [48] (Additional Files 28 and 29). *VviERF6L* signal intensity peaked significantly at pre-veraison and generally declined as berries approached full ripening (FR) (Additional File 28). There were subtle changes in signal intensity level over the years and vineyards (Additional File 29), but generally expression levels were similar and the *VviERF6L* expression pattern remained conserved, indicating these genes may not be strongly influenced by environment during berry development.

### *VviLISCL3* and *VviCML45* were the most connected genes to the *VviERF6Ls*

Two genes share a similar expression pattern as the 18 *VviERF6Ls* across various cultivars, organs, and treatments. The meta-data analysis was completed with a gene co-expression analysis to identify genes sharing expression patterns for all *VviERF6Ls* as a clade between the five data series that were re-analyzed with the V3 annotation of PN40024 (SRP117281, PRJNA516950, GSE67191, GSE62744, and GSE62745). The top 100 genes most connected to each *VviERF6L* were extracted from the TOM (Topological Overlap Matrix) for each WGCNA. Common co-expressed genes were identified by comparing these sets of genes. In total, two genes were identified in all five data series that were the most connected to the *VviERF6L* clade across the various conditions and variables of each data series (Additional File 30). The two co-expressed genes were a Scarecrow-like transcription factor, *VviLISCL3* (Vitvi06g00489), and a calmodulin-like protein, *VviCML45* (Vitvi14g01975). There were several other genes that shared expression patterns with the *VviERF6L* clade in four out of the five data series including *VviERF1* and *VviWRKY33* (Additional File 31). The full list of genes co-expressed with the *VviERF6L* clade in four of the five data series is in Additional File 31. Six of the 16 genes sharing the *VviERF6L* expression pattern were unannotated.

After analyzing the *VviERF6L* clade as a whole, the co-expression analysis was repeated for each *VviERF6L* individually. Surprisingly, no gene was connected to any *VviERF6L* in all five data series in this individual analysis, not even the other *VviERF6Ls*. Because no genes were co-expressed with any *VviERF6L* individually, genes co-expressed in four out of the five RNA-Seq data series were considered. *VviWRKY33* was the only gene to be co-expressed in four out of the five data series in this individual *VviERF6L* gene co-expression analysis but only for *VviERF6L11* and *16*. The low number of genes co-expressed with *VviERF6Ls* in all five RNA-Seq series (two for the clade co-expression analysis (Additional File 31) and zero for the individual *VviERF6L* co-expression analysis) may be a result of the diverse RNA-Seq series utilized that examined various organs, genotypes, developmental stages, and stresses.

### Summary of the meta-analysis

**Fig. 12:**
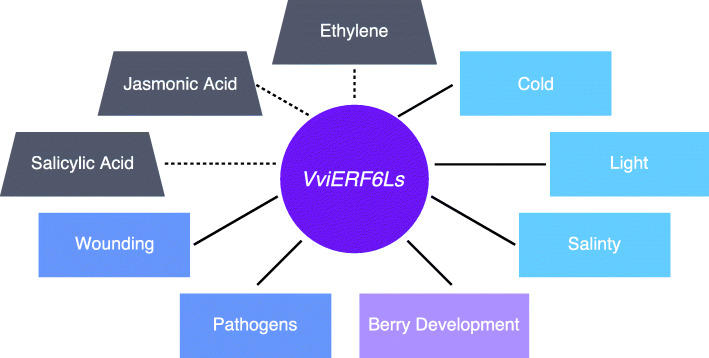
*VviERF6L* clade transcriptional response model. A summary model of conditions *VviERF6Ls* transcriptionally responded to in the meta-data analysis are connected with solid lines. Conditions linked to those investigated in the meta-data analysis requiring further confirmation are connected with dotted lines. Phytohormones are shown in grey. Developmental stages are shown in purple. Abiotic stresses are shown in light blue, and biotic stresses are shown in darker blue

Generally, *VviERF6Ls* were lowly expressed in all data sets, but these genes demonstrate striking fold changes in expression levels and significant differential expression under numerous conditions. *VviERF6Ls* are broadly expressed across grapevine organs, tissues and in response to various abiotic and biotic stresses as well as throughout berry development (Fig. 12). *VviERF6Ls* appear to increase in expression in response to severe water deficit and salinity. However, over more long-term moderate water deficit, *VviERF6Ls* are generally decreased. *VviERF6Ls* have distinct differential expression in response to cold and light in different cultivars. *VviERF6Ls* are differentially expressed in response to various pathogens, but the level of expression varies depending on the pathogen and cultivar. *VviERF6Ls* are also differentially expressed across berry development with the highest expression levels as berries transition into veraison. *VviERF6L* expression patterns are highly conserved across cultivars, vineyards, and years. The broad range of *VviERF6L* expression across tissues and expression patterns are conserved throughout numerous data series. The transcriptional response for each member of the *VviERF6L* clade was dependent on a number of factors (organ, time, treatment, duration, severity, genotype, etc.). The transcriptomic response for each experimental design, while generally conserved, was unique for each *VviERF6L* (i.e. some members of the clade increased in transcript abundance, some decreased, and others did not respond under a specific condition). The individuality emerging in the *VviERF6L* clade as well as divergent observations for different severities of similar treatments makes it difficult to generalize common responses. However, a diagram (Fig. 12) was constructed to summarize the conditions that elicited *VviERF6Ls* responses as well as conditions requiring additional data to further elucidate the role *VviERF6Ls* play in grapevine. As transcriptomic technology evolves, the *VviERF6Ls* will be able to be better differentiated and understanding of the clade will be improved.

Two results are clear, first, in all the data series discussed, *VviERF6L12,* one of the truncated *VviERF6Ls*, repeatedly demonstrated significantly higher expression than any of the other *VviERF6Ls. VviERF6L12* had 2–228 times more transcript abundance than the average of all other *VviERF6Ls*. Across the treatments and conditions of the selected data sets, the transcript abundance of *VviERF6L12* averaged 25.6-times more RMA-normalized signal intensity, counts, FPKM, or TPM than the average expression of the other *VviERF6Ls* (Additional File 32). Second, *VviERF6L8* was frequently the lowest expressed *VviERF6L* with no detectable expression in certain cultivars. The vast range of *VviERF6L* expression levels made it necessary to log_2_ transform the data in the meta-data analysis, so each *VviERF6L* expression was visible. Although the *VviERF6L* clade is highly conserved, each *VviERF6L* is under unique transcriptional regulation.

### Overexpressing *VviERF6L*1 in *Vitis* had minor impact on the transcriptome and phenotype

In previous microarray studies, *VviERF6L1* appeared to be the most responsive *VviERF6L*, with transcript abundance increasing in CS in response to severe leaf dehydration [17] and with changing sugar levels in a study of the late stages of berry ripening [16]. Further investigation of *ERF6L1* function was investigated with *VviERF6L1* overexpression and knockdown lines. Attempts to establish *Vvierf6l1* knock-down lines failed; plants were unable to be re-established after transformation with a T-DNA insertion. An empty vector control (G1) and *VviERF6L1* overexpression lines (L12–1, L12–2, L12–3, L12–11, and L12–23) were created in a Seyval Blanc background under the control of a bi-directional duplex 35S promoter fused to EGFP/NPTII in pECBC [49]. Overexpression was confirmed with semi-quantitative PCR and RT-qPCR to verify stable over-expression (Additional File 33). Extensive phenotyping revealed *VviERF6L1* overexpression lines did not exhibit a morphological phenotype under control conditions, or in response to salinity, water deficit or pathogen spread treatments (Additional File 34). Potential downstream targets of *VviERF6L1* were determined with differential expression analysis on RNA-Seq data from leaves of the empty vector control (G1) and *VviERF6L1* overexpression lines (L12–3, L12–11, and L12–23). *VviERF6L1* was the only *VviERF6L* gene with significantly higher expression in the overexpression lines compared with G1 (Additional File 35). In total, only 14 genes were significantly differentially expressed in all three overexpression lines relative to G1 (Additional Files 36 and 37). Up regulated genes included: *VviERF6L1* (Vitvi16g00350), *CYP722A1* (Vitvi04g01352), *CRK8* (Vitvi11g01160), *LAC14* (Vitvi18g01479). Down regulated genes included: three *PRB1* (pathogenesis-related protein 1) genes (Vitvi03g00757, Vitvi03g01649, Vitvi03g01651), unannotated genes (Vitvi03g01650, Vitvi07g01985, Vitvi11g01692, Vitvi18g02319), *MET1* (Vitvi12g02119), *WAKL1* (Vitvi18g00024), and *LIMYB* (Vitvi01g01444).

## Discussion

### The *VviERF6L *clade was expanded and conserved

*VviERF6L* genes with no previously known functions were identified to be an expanded clade in *Vitis* in comparison with other plant species. Using protein and promoter motifs and a meta-data analysis, this work shows *VviERF6Ls* are highly conserved proteins. Manual and *in silico* techniques identified and confirmed 18, 26, 15, and 14 members of the *VviERF6L* clade in PN40024, CS, CH, and CA respectively (Additional File 12). The high sequence and spatial conservation of amino acid motifs validates these sequences as members of the *VviERF6L* clade in the AP2/ERF subfamily IX. The small differences in protein motif sequence and position may contribute to the differential regulation of the *VviERF6Ls* observed in the publicly available microarray and RNA-Seq analysis. Four known protein domains identified (Fig. 1) in the VviERF6L proteins coincide with those in Arabidopsis ERF IXb transcription factors including ERF5 (At5g47230), ERF6 (At4g17490), ERF104 (At5g61600) and ERF105 (At5g511290) [15]. The VviERF6Ls contain CMIX-2, 5, and 6 domains (putative post-translational modification sites) as well as the AP2/ERF domain (DNA-binding) (Fig. 1). While VviERF6L1 has highest orthology to AtERF6, it contains an additional domain, CMIX6, found in AtERF104, but not present in AtERF6. This domain is thought to contain a MAP kinase phosphorylation site [15].

Four motifs located near the amino and carboxyl ends of the VviERF6L proteins were unable to be identified. ERF transcription factors interact with numerous other proteins including regulatory enzymes, coactivators, repressors, and other transcription factors [50–52]. These interactions regulate stability and activity as well as localization of ERFs. The unidentified VviERF6L protein domains may play roles in post-translational regulation and/or interactions with other proteins. One such interaction occurs with AtERF104, which is phosphorylated by MPK6 and released from this interaction in the presence of the flg22-peptide to influence ethylene signaling and pathogen susceptibility [53].

*Vitis vinifera* had the highest number of *VviERF6L* paralogs compared to species with the closest related genes (Additional File 5), marking this as an expanded clade in grapevine. The *VviERF6L* clade consists of nearly consecutive genes on chromosome 16. The *VviERF6L* paralogs likely originate from gene duplications. Duplication events are common and frequent in plants contributing to gene evolution and diversification [54]. Recent whole genome duplications in cotton [55], wheat [56], and soybean [57] and dispersed duplication in corn [58] gave rise to agronomically valuable traits of 4 of the top 10 produce crops in the United States. Exploring *VviERF6L* evolution in wild grapevine as well as the ancestor of domesticated grapevine may provide more evidence and a time line for the hypothesized duplication event. The contiguous *VviERF6Ls* may be tandem array genes, arising from tandem duplication of an ancestral gene. The significance of tandem ERF duplication was recently described in *Fragaria vesca* [59]. Tandem *Fve*ERFs are differentially expressed from one another in response to abiotic stress, suggesting gene divergence occurring after tandem duplication(s) [59]. A similar event may have occurred with grapevine *VviERF6Ls*. The possibility of *ERF6L* duplication in *Vitis* is supported by the expansive ERF family in pear (155 members) [60], another woody perennial, as well as *Arabidopsis* (122 members) and rice (139 members) [15].

### Individual *VviERF6Ls* had unique gene expression

The *VviERF6L* clade is under unique transcriptional regulation in response to numerous conditions (Fig. 12). Contrasting the high similarity in protein sequence, *VviERF6L* putative promoter regions (Fig. 3) showed greater diversity than VviERF6L protein sequences (Fig. 1), indicating these genes are under distinct transcriptional regulation. However, strong patterns and conserved motifs were detected across the promoter regions. The CAATBOX1 motif was the most abundant motif in the 18 *VviERF6L* upstream regions (Additional File 15), which may play a role in tissue specific gene expression [61] and contribute to *VviERF6L* expression across the broad range of tissues observed in the meta-data analysis. The MYB1AT motif is present in dehydration responsive genes like *RD22* [62]. This motif was present in all *VviERF6L* promoters, but with fewer repetitions, supporting the transcriptomic data that *VviERF6Ls* are only responding to severe water stress (Fig. 5). DOFCOREZM, another abundantly present cis-regulatory element in the *VviERF6L* promoter regions (Additional File 15), is the binding motif for Dof proteins, a diverse group of transcription factors with roles in defense and phytohormone responses, light, and development [63]. Interestingly, numerous *VviERF6L* promoter motifs associated with light responses were identified including the CACTFTPPCA1, DOFCOREZM, GATABOX, GT1CONSENSUS, with responses supported by the results from the meta-data analysis (Fig. 8 and Additional File 26). AtERF5, has also been linked to light responses [64]. It is possible the light response of *VviERF6Ls* is tissue specific and was not well identified in the berry-centric microarrays and RNA-Seq data sets, requiring further investigation in other tissues. Several motifs associated to biotic stress responses were also present in the promoter including WBOXATNPR1, which had the highest number of repeats in *VviERF6L12*. Together, the presence of these motifs supports the proposed roles of *VviERF6Ls* in extreme water deficit, cold, light, and pathogen responses.

The diverse promoter sequences partially explain the distinct *VviERF6L* expression levels and patterns observed across the RNA-Seq and microarray series. *VviERF6L12* had one of the most variable promoter sequences (Additional File 16) along with *VviERF6L1* and *VviERF6L6*. Putative promoter regions of *VviERF6L12* (and other *VviERF6Ls*) contained unique cis-regulatory elements as well as distinctive motif placement and replication. The distinct conditions in which certain *VviERF6Ls* are DEGs (Additional File 23) in the meta-data analysis may be partially explained by the diversity in upstream sequences.

Additional differences in transcriptional regulation may be contributed to by epigenetics. ERFs have been demonstrated to regulate one another and effect epigenetic regulation through the ethylene-responsive element binding factor-associated amphiphilic repression (EAR). The EAR motif is a short peptide sequence comprised of charged and polar residues (LxLxLx or DNLxxP) that are proposed to confer gene silencing via histone modification and chromatin remodeling through an unresolved mechanism [2, 65]. AtERF3, 4, and 7 contain EAR motifs that act as active repressors of target and reporter genes [66–69]. Epigenetic regulation and upstream effectors of *VviERF6L* expression require further investigation to determine interactions with EAR containing ERFs.

### *VviERF6L1* expression was independent of ABA treatment

The transcript abundance of *VviERF6Ls* was shown to increase in response to severe osmotic stress and at pre-veraison in berry develop in preliminary microarrays that originally brought attention to the *VviERF6L* clade [16, 17]. Although ERFs are traditionally associated with ethylene signaling, these ERF transcription factors are well documented to act as hubs for hormone-crosstalk and signaling integration [9–14]. ABA is a key phytohormone in abiotic stress responses and berry ripening in grapevine. Water deficit responses are ABA-dependent and/or ABA-independent [70]. However, *VviERF6L1* expression was not significantly different in CS leaves treated with exogenous ABA relative to control (Additional File 21). Interestingly, *AtERF5* is also not associated with ABA signaling [71]. It is possible other *VviERF6L* transcripts may increase or decrease in response to ABA treatment, as the *VviERF6Ls* are distinctly regulated, but no known ABRE cis-regulatory elements were identified in the *VviERF6L* upstream regions (Additional File 15). Alternatively, a *VviERF6L* ABA response may be tissue specific (e.g. berries transitioning into veraison). The increased expression of *VviERF6L1* in response to various abiotic stresses and berry development in the meta-data analysis, but the lack of induction in response to ABA foliar spray indicates the *VviERF6L1* abiotic-stressed-based induction may be independent of the ABA signaling pathway. RT-qPCR along with microarrays and short-read RNA-Seq were determined to be less than ideal techniques for quantifying *VviERF6L* transcripts due to the high sequence similarity of these genes; primers and probes could hybridize to (and short reads could be attributed to) multiple *VviERF6Ls* resulting in inflated transcript levels and difficulty separating the *VviERF6Ls* independently. With the advent of Iso-Seq and the ability to quantify full-length transcript reads, the future of distinguishing *VviERF6Ls* individually will be more reliable and accurate [72].

### *VviERF6Ls *were differentially expressed in response to water deficit, salt, and cold

*VviERF6L* expression significantly increased in response to extreme water deficit and salt (Figs. 4 and 5). However, exposing CS, RA, RI, and SC vines to a moderate one- and two- week natural dry down revealed *ERF6Ls* were generally significantly decreased in transcript abundance (Fig. 6). Each *VviERF6L* had a unique expression level that responded differently in each species and organ examined in this experiment, further distinguishing the *VviERF6Ls* individually and supporting the hypothesis each *VviERF6L* is under specific transcriptional regulation.

The uniqueness of *VviERF6Ls* across tissues and cultivars is further demonstrated with significant differential *VviERF6L* expression in response to cold (Fig. 7 and Additional File 24 and 25). Again, each *VviERF6L* in the different cultivars had varied responses to the cold (Fig. 7). There were also differences between the leaves and shoot tips investigated in the different data series. *VviERF6Ls* generally followed similar expression patterns within treatments but to different degrees of expression across cultivars and *Vitis* species indicating differential *ERF6L* regulation across these division of *Vitis*. Differences in *ERF6L* regulation may contribute to differences in abiotic stress tolerance across various grapevine cultivars.

### *VviERF6Ls* were differentially expressed in response to biotic stresses

Differential expression analysis performed on *VviERF6L1 Vitis* overexpression relative to control vines revealed three *PRB1* paralogs, one putative *PR1*, and a putative mildew resistance locus that were significantly down regulated in the overexpression lines. *PR1* is a common SA signaling marker gene up-regulated in response to certain pathogens including *Pseudomonas syringae* [73–76]. The distinct downregulation of this gene and its paralogs in *VviERF6L1* overexpression lines is consistent with the enhanced susceptibility to *Pseudomonas syringae* documented in the *AtERF6* and *AtERF5* overexpressors [77]. It is possible VviERF6L1 works in combination with other TFs to impact grapevine susceptibility to various pathogens. Another DEG in OX *VviERF6L1* lines, *LAC14*, may also be linked to biotic stress response. This gene encodes a laccase that is part of secondary metabolism responsible for lignin degradation or polymerization [78] and was significantly upregulated in the OX *VviERF6L1* lines. Lignin biosynthesis and accumulation aids in plant resistance to insect pests. Lignin deposition is also associated with abiotic stress response and antagonization with plant growth [79, 80]. The duality of the DEGs’ roles in both abiotic and biotic stress response further strengthen the hypothesis of broad functionality of *VviERF6Ls*. *VviERF6Ls* were differentially expressed in response to various pathogens in the meta-data analysis (Figs. 9, 10 and Additional File 27). The level of *VviERF6L* expression and specific *VviERF6Ls* that were DEGs was pathogen and tissue specific.

*VviERF6Ls* response to pathogens is conserved in Arabidopsis. AtERF5, one of the closest orthologs to the VviERF6L clade, directly interacts with AtERF6 and 8 as well as SCL13 and MPK3 and 6 in unique combinations to respond differentially to *Pseudomonas syringae* [81], *Botrytis cinera* [77], *Alternaria brassicicola* [81]*,* and *Meloidogyne incognita* [82]. *Aterf5/6* double mutants have enhanced susceptibility to *V. longisporum*, a fungus that induces severe wilting and plant death [83]. Another pathogen related example of a potential role in pathogen response shows decreased *VviERF6L* expression in response to *Plasmopera viticola* (downy mildew) in more susceptible vines and either an increase or no significant change in transcript abundance in more tolerant vines [84, 85].

### *VviERF6Ls* were differentially expressed over the course of berry development

The *VviERF6Ls* had a consistent significant differential expression pattern over the course of berry development. *VviERF6L* transcript abundance was significantly increased at pre-veraison as the berries transitioned into ripening and decreased as the berries approached full ripening and harvest (Fig. 11). The *VviERF6L* expression pattern over berry development was conserved across red and white berries (Fig. 11), vineyards, and years (Additional Files 28 and 29).

The *VviERF6L* ortholog *Solyc08g078190* identified from the Pan-taxonomic Compara Gene Tree in Gramene follows a similar expression pattern as *VviERF6Ls.* This gene, annotated as SlERF.B13 [86] or ERF1a [87], increases in the breaker stage (equivalent to veraison in grapevine) of berry development in tomato and decreases in transcript abundance as berries ripen [86]. Another *VviERF6L* tomato ortholog, *Sl-ERF.B3* (Solyc05g052030), also plays a role in berry development [88]. Like grapevine, tomato *ERFs* can have increased or decreased transcript abundance under certain conditions [86]. This similarity between non-climacteric grapevine and climacteric tomato along with the significant transcriptomic responses in the meta-data analysis support a potential role of *VviERF6Ls* in berry development requiring further investigation.

### *VviLISCL3andVviCML45* were genes connected to the *VviERF6L *clade

Little is known about *VviLISCL3* and *VviCML45* that were co-expressed with the *VviERF6L* clade. VviLISCL3 is a GRAS transcription factor with roles in plant development, abiotic stress, and disease response [89, 90] similar to the *VviERF6L* expression profile identified from the meta-data analysis. *VviLISCL3*, like the *VviERF6Ls*, appears to be ubiquitously expressed across plant tissues with the exception of pollen [91]. *VviLISCL3* was differentially expressed over berry development and likely plays a role in berry set and the early stages of berry development [91]. However, unlike the *VviERF6Ls*, *VviLISCL3* had high expression levels at ripe, harvest, and post-harvest stages of berry development [91]. *SlGRAS13*, the *VviLISCL3* ortholog in tomato, shows the same expression pattern and role in fruit ripening [92]. *AtCML45*, the Arabidopsis ortholog of *VviCML45*, is differentially expressed in *nrp1 nrp2* Arabidopsis double mutants that lack these histone chaperones associated with root growth [93] and may provide a very loose link of VviERF6Ls to epigenetic modification that ERFs are known to play a role in [2, 65]. The link between VviERF6Ls, VviLISCL3, and VviCML45 was discovered but remains unresolved, requiring further clarification.

### Overexpressing *VviERF6L1* had a minimal impact on grapevine

Overexpressing *VviERF6L1* in *Vitis vinifera* did not result in a detected morphological phenotype. This observation may be attributed to the limited number of genes *VviERF6L1* overexpression affected (Additional File 30). It is possible a *VviERF6L1* overexpression phenotype is only detectable under specific conditions not tested in this work. *VviERF6L1* was the only member of the *VviERF6L* clade with enhanced gene expression in the *VviERF6L1* overexpression lines (Additional File 35). The other *VviERF6L* genes may share similar functions and could have been down regulated in response to the overexpression of *VviERF6L1*. The hypothesis that *VviERF6L* genes share similar functions is supported by the redundant gene and promoter sequences of *VviERF6L* genes (Figs. 2 and 3). Paralog downregulation in response to overexpression is observed in plants. For example, *CYP78A8*, one of the closest paralogs to *CYP78A9*, is downregulated in response to *CYP78A9* overexpression [94]. However, this does not appear to be the case in the overexpression of *VviERF6L1.* The promoter and meta-data analysis support this conclusion. Although the *VviERF6Ls* share similar expression patterns, expression levels and transcriptional regulation are unique for each *VviERF6L*, and the overexpression of *VviERF6L1* does not appear to influence the transcription of the other *VviERF6Ls*. Further studies are needed to identify and confirm specific VviERF6L downstream targets.

### VviERF6L in grapevine is distinct from Arabidopsis ERFs

*Arabidopsis thaliana ERFs* do not have strong orthology to *VviERF6Ls*. AtERF5 and AtERF6 are the closest orthologs to VviERF6L1. *AtERF6* and *AtERF5* are rapidly induced in growing tissues and effectively arrest cell cycle progression and plant growth in response to osmotic stress [95]. *AtERF5* is involved in karrikin signaling [71], water deficit and osmotic stress [96], programmed cell death [97], and immunity response [77]. *AtERF6* overexpression lines are hypersensitive to osmotic stress [96]. *VviERF6L1* overexpression vines exposed to chilling, water deficit, and salinity demonstrated no significant differences from controls in the reduction in growth, carbon assimilation, or canopy surface area relative to empty vector control plants (Additional File 34). However, *VviERF6Ls* were shown to respond transcriptionally to osmotic stress (Fig. 5), but no link was made to cell cycle regulation. AtERF6 is also a positive regulator of antioxidant production with *Aterf6* mutants having stunted growth and enhanced levels of ROS and anthocyanin content [98]. AtERF6 is an inductor of stress tolerance genes and a key activator of leaf growth inhibition [99]. *VviERF6L1* overexpression lines had no reduction in growth or development relative to empty vector controls (Additional File 34), and no connection was made specifically to antioxidants and anthocyanins.

In Arabidopsis, *AtERF6* acts as a regulatory hub favoring stress defense mechanisms at the cost of plant growth through DELLA protein stabilization via ethylene and gibberellin crosstalk [99, 100]. *VviERF6Ls* were found to respond to various pathogens (Figs. 10, 11 and Additional File 27), but no negative impact on growth was found at least in the case of *VviERF6L1* (Additional File 34). It is possible other *VviERF6Ls* could impact growth. *VviERF6L1* overexpression lines did, however, have significantly decrease transcript abundance of several *PR1B* genes, associated with pathogen stress response (Additional File 36). AtERF6 and AtERF5 function redundantly in response to biotic stresses and act as a point of crosstalk between ethylene and JA signaling, providing enhanced resistance to *Botrytis cinera,* but increased susceptibility to *Pseudomonas syringae* in *AtERF5* and *AtERF6* constitutive plants. *Aterf5/Aterf6* double mutants demonstrate enhanced susceptibility to *Botrytis cinera* [77]. Preliminary pathogen spread assays did not show significant differences in OX *VviERF6L1* leaves relative to empty vector control (Additional File 34). Further investigation of *VviERF6L1* overexpression susceptibility to various pathogens is on-going and may reveal a more definite role in biotic stress response.

*AtERF6* overexpression lines demonstrate extreme dwarfism [96]. AtERF6 activates *AtERF11* transcription, which in turn competes with AtERF6 for DNA-binding sites as a balancing mechanism between stress response and growth [99]. *AtERF11* overexpression is able to rescue the dwarf phenotype in *AtERF6* overexpression plants [99]. A VviERF6L1-ERF11 antagonism was not detected in the DEA of the *VviERF6L1* overexpression lines. It is possible a different VviERF6L is responsible for this regulatory mechanism or that this interaction is not present in *Vitis*.

*AtERF6* is also documented to activate *MYB51*, *WRKY33*, and *STZ* [96], all genes with roles in biotic [101, 102] and abiotic [103–105] stress responses. *VviERF6Ls* are co-expressed with *VviWRKY33* (Additional File 31), linking the two species in this signaling pathway, but at least *VviERF6L1* does not appear responsible for *VviWRKY33* activation (Additional File 34). The distinction of the *Vitis* ERF6L clade from the closest *Arabidopsis* orthologs is supported by the work presented here including the lack of comparable phenotypes in overexpression lines and transcriptomic responses from the meta-data analysis. However, while *Vitis ERF6L* genes are unique, they may functionally overlap with the distant Arabidopsis orthologs to an extent with associated abiotic and biotic stress responses. ERF TFs in Arabidopsis and *Vitis* are differentially regulated by abiotic stresses [106] including cold, salinity [107], and water deficit as well as biotic stresses such as wounding and pathogen attack [108, 109].

## Conclusions

*VviERF6Ls* are an expanded and highly conserved *Vitis* clade. *VviERF6L* expression was increased in berries at the pre-veraison stage and was found to be induced in leaves by extreme abiotic stress including salt, cold, and water deficit (Fig. 12). *VviERF6L1* was not induced by ABA, indicating a role in water deficit responses through an ABA-independent pathway. Overexpression of *VviERF6L1* in a Seyval Blanc background did not yield a detectable morphological phenotype, emphasizing the separation of this clade from the Arabidopsis orthologs *ERF6* and *ERF5*, overexpression of which results in extreme dwarfism and osmotic stress sensitivity. DEA performed on RNA-Seq from the *VviERF6L1* overexpression lines identified 14 DEGs involved in abiotic and biotic stress responses. Overall, *VviERF6Ls* have versatile functions and are expressed in numerous tissues in response to abiotic and biotic stress and may play multiple roles in these processes that require further elucidation.

## Methods

### Phylogenetic analysis and cis-regulatory element identification of the ERF6-like clade

DNA sequences from the *VviERF6-like* clade and *Arabidopsis thaliana* orthologs were obtained from ORCAE and Araport11 and compared to CRIBI and TAIR identifiers respectively [18, 110–112]. Sequences were aligned with MUSCLE using the msa R package and a phylogenetic tree was drawn using a clustal omega alignment in Mega X [31, 113]*.* A Maximum likelihood method and Jones-Taylor-Thornton matrix-based model with Bootstrapping *n* = 1000 replicates were used to create a consensus tree consisting of PN40024, CS, CH, and CA VviERF6Ls and PN40024 ERFs. Branches present in < 50% bootstrap replicates are collapsed. The initial tree for the heuristic search was generated from the maximum parsimony method. The Subtree-Pruning-Regraftings - Fast (SPR level 3) was used. Conserved motifs were identified and confirmed in MEME using the standard settings (motif limit at 50 amino acid residues) [22, 114]. Motifs were characterized with InterPro and modeled in SWISS-Model using standard settings [24, 25]. *VviERF6L1* gene upstream regions (− 3000 bp) were obtained with the R package GenomicFeatures [115]. Cis-element enrichment and identification analysis was performed with PLACE [32]. Promoter regions were aligned, and a phylogenetic tree was made in clustal omega.

### Meta-data analysis

RNA-Seq and microarray data sets were downloaded from NCBI GEO [35] and SRA [36] with GEOquery [116] version 2.50.5 and the SRA Toolkit version 2.9.2, respectively. Data series that were re-analyzed with the V3 PN40024 annotation were quality checked with fastqc [117] and trimmed with trimmomatic version 0.35 [118]. Transcript abundance was quantified with Salmon version 0.10.1 [119] using quasi-mapping, seqBias, gcBias, fldMean 50, fldSD 1, validateMappings, libType A, and rageFactorizationBins 4. Tximport version 1.10.1 was used to generate the count matrix. Differential expression analysis was performed with DESeq2 version 1.22.2 [120]. Co-expression analysis was performed using WGCNA version 1.68 for all 18 *VviERF6Ls* as a clade as well as for each *VviERF6L* individually using the five data series that were re-analyzed with the V3 annotation of PN40024. The top 100 genes most connected to the *VviERF6L* clade was used to make a Venn diagram (Additional file 30).

### Plant transformation

*VviERF6L1* CDS was inserted into pECBC under the control of a bi-directional duplex 35S promoter fused to PR1 and EGFP/NPTII [49]. Seyval Blanc cell cultures were transformed with *Agrobacterium tumefaciens* and grown under kanamycin selection. The empty vector was also inserted into cells to be used for control plant generation. Transgenic lines were created at the Mid-Florida Research and Education Center for the Institute of Food and Agricultural Sciences.

Transgenic cells were confirmed with GFP screening performed with confocal microscopy. Four overexpression lines (L12–1, L12–2, L-12-3, L12–11, and L12–23) and one control line (G1) were regenerated into full plants grown under greenhouse conditions.

### Plant materials and growth conditions

Own-rooted *Vitis vinifera* (L.) cv. Cabernet Sauvignon clone 8 (CS) were obtained from Inland Desert Nursery (Benton City, Washington, USA). *Vitis champinii* cv. Ramsey (RA), *Vitis riparia* cv. Riparia (RI), and *Vitis vinifera x girdiana* SC2 (SC) were obtained from the Plant Foundation Services at UC Davis (Davis, CA USA). Mature plants of the five transgenic lines and of the four genotypes CS, RA, RI and SC were grown in Stuewe and Son’s tree pots TP915R (22.9 cm × 39.4 cm) containing 1:1:1:2 perlite:peat moss: Grow Mulch (Kellogg):washed sand. Each pot contained ~ 8.0 kg of soil mix. Mature plants were irrigated with ~ 1.2 L of pH 5.5 water bi-weekly. Propagates were generated from single node cuttings of mature plants and transferred in trays containing pH 5.5 water with an air-stone until roots emerged. Plants were transferred to Stuewe and Son’s Anderson AB39 pots (7.3 cm × 22.9 cm) consisting of ~ 1.0 kg quikrete medium grain sand and ~ 40 g of 50:50 perlite-vermiculite mix. Plants were covered for 1 week to increase relative humidity and slowly acclimated to greenhouse humidity conditions over the course of 2 weeks. Greenhouse conditions were maintained at approximately 21–26.5 °C and 20–50% relative humidity. All pots were elevated 7.5 cm off the floor with perforated black plastic flats. Light was supplemented with 1000 W high pressure sodium light bulbs approximately 4.5 m above the floor directly over the center of the experimental area. Supplemental light had a 16:8-h light-dark cycle. Light intensity of the greenhouse averaged 1200 μE m^− 2^ s^− 1^. Propagates were irrigated every other day (until they reached approximately 70 cm height at which point the experimental treatment began) with Cramer’s complete nutrient solution (1.5 mM Ca (NO_3_)_2_, 2 mM KNO_3_, 0.6 mM MgSO_4_, 1 mM KH_2_PO_4_, 1.5 mM CaCl_2_, 36 μM Fe_2_^+^ Sprint 330, 1 μM MnSO_4_, 0.5 μM CuSO_4_, 20 μM ZnSO_4_, 20 μM H_3_BO_3_, and 0.01 μM (NH_4_)_6_Mo_7_O).

### Phenotypic characterization of *VviERF6L1* overexpressing lines

Mature transgenic plants were grown as a single shoot. Weekly measurements were taken for stem length, number of nodes, internode length, number of leaves, leaf length (from petiole attachment point to the tip of the leaf down the midvein), leaf width (from one side of the leaf to the other at the widest point perpendicular to the midvein), leaf lobe sinus lengths and angles, leaf surface area, tendril emergence, and berry development (berry occurrence and circumference). Stem elongation rate was calculated from repeated stem length measurements. Leaf surface area was obtained from photographs using ImageJ version 1.52 [121]. Leaf measurements were performed weekly on at least ten plants per line and repeated continuously over at least 6 months. All measurements were performed on similar nodes to ensure uniform developmental stages. Shoots were pruned when the plant height reached 1 m, at which time, measurements were repeated as a new shoot emerged at the cane. Leaf length measurements were repeated over the course of 3 years. To phenotype roots, overexpression line propagates were transferred to 12 L hydroponic tubs containing an air-stone and 0.5x strength Cramer’s complete nutrient solution when roots were ~ 5 cm. Propagates were placed in tight fitting lids and allowed to grow for 20 days under greenhouse conditions. Roots were imaged and analyzed with WinRHIZO every 5 days. Measurements included total root length, total root surface area, number of primary lateral roots, number of adventitious roots, and plant fresh weight. Two mature leaves at similar developmental stage from each transgenic line from three individual cloned plants were excised from mature plants and frozen in liquid nitrogen for RNA sequencing. Berry occurrence, number and circumference were photographed and quantified with ImageJ.

### Abiotic stress and hormone treatments

Treatments consisted of control treatment that entailed irrigating plants daily with 100 mL complete nutrient solution under greenhouse conditions (control); a salinity treatment: that was irrigating plants daily with 100 mL complete nutrient solution with 100 mM NaCl and 20 mM CaCl_2_ added; a cold treatment: that was growing plants in a 10 °C growth chamber with a light intensity 50 μE and irrigating daily with 100 mL 10 °C complete nutrient solution; a water deficit treatment: that was maintaining pots at a low 30% relative water content. Water deficit pots were dried down to 30% relative water content by withholding irrigation at which point they were maintained daily at 30% pot relative water content, for 1 and 2 weeks. Control plants were watered in excess daily. After 20 days of salt, cold and control treatment, four experimental replicates of individual G1, L12–1, L12–2, L12–3, L12–11, and L12–23 vines were harvested. After 1 and 2 weeks of control and water deficit treatment, five experimental replicates of G1, L12–1, L12–2, L12–3, L12–11, and L12–23 individual vines were harvested. Shoot, stem, leaf, and root fresh and dry weights were measured in addition to total canopy surface area measured from photographs with ImageJ.

To examine *VviERF6L1* response to hormones, CS leaves were sprayed with 10 μM ProTone (s-ABA) (Valent BioSciences LLC) or water (control) for 1 h. All sprays contained 0.5% Tween20. For spray treatments, mature leaves were selected and sprayed to saturation (solution dripping from leaves) on both sides of the leaf [122]. All samples were frozen in liquid nitrogen for all treatments. These experiments were performed in triplicate with each round consisting of three individual leaves of similar developmental stage from separate plants per genotype per harvest-time. Different sprays were made for each round.

Chilling treatments were performed on CS, RA, RI, and SC plants placed in a 4 °C growth chamber with a light intensity of ~ 200 μmol m^− 2^ s^− 1^ in a randomized-block experimental design. Eight thermometers were placed evenly throughout pots in the growth chamber. Pot temperature was recorded before each harvest time. Control plants were kept under greenhouse conditions. Total leaves were harvested within 2 min after 2 h of chilling treatment and frozen in liquid nitrogen. These experiments were performed in triplicate with each round consisting of three individual plants per genotype per harvest-time per treatment. Additional chilling treatments were performed in a 4 °C refrigerator. RA and CS leaves of comparable age and size were placed on a wire support in pre-chilled or control Tupperware boxes containing 200 mL DI water similar to what has been described previously [123]. Petioles were placed in the water and lamina was supported by the wire rack above the water. A light-proof cardboard box was placed over the leaf-containing Tupperware box to prevent light intrusion. Control samples were placed under a light-proof box at 23 °C. Leaves were harvested within 2 min after 2 h of temperature treatment and frozen in liquid nitrogen.

### RNA extraction

All samples were ground with a mortar and pestle under liquid nitrogen. RNA extraction for RNA-Seq samples was performed with a CTAB-based method including an RNase-free DNase treatment as previously described [124]. RNA-Seq samples were prepared from 1.3 μg RNA. Quality and concentration were confirmed with Ribogreen technology performed by the Nevada Genomics Center and Experion RNA StdSens Chips (Bio-Rad).

RNA from leaves of the plants treated with either abiotic or hormone treatment was extracted with a Spectrum Total Plant RNA kit (Sigma-Aldrich) modified protocol [125]. All RNA extractions were treated with RNAse-free DNase I (Qiagen) to remove genomic DNA contamination. RNA concentration, quantity, and purity for all samples was confirmed with a Nanodrop spectrophotometer, a 1.2% quality gel loaded with 400 ng RNA from each sample, and a 2% gel loaded with 10 μL LAR intron PCR product. LAR PCR products were amplified from a 10 μL GoTaq Green Master Mix (Promega) containing 250 ng RNA and 0.5 μM forward and reverse primers specific for a LAR intron. The PCR reaction included 95 °C for 2 min, 35 cycles of 95 °C for 30 s, 62 °C for 25 s and 72 °C for 25 s. Purified samples demonstrating two bands corresponding to ribosomal subunit RNA, no band corresponding to the LAR intron, and sufficient concentration were utilized for RT-qPCR.

### PCR and RT-qPCR

All samples from cold and hormone spray experiments were reverse transcribed with iScript Reverse Transcriptase Supermix (Bio-Rad) according to manufacturer’s instructions from 2 μg RNA. Primers were designed using NCBI Primer-BLAST. Primer sequences are provided in Additional File 38. Primer efficiencies were verified on purified PCR products (Machery-Nagel NucleoSpin® Gel and PCR Clean-up kit) and will be considered at 100% for the gene expression calculations. All reactions were performed on a Bio-Rad Real-Time thermal cycler CFX96 with the following protocol: 95 °C for 3 mins; 40 cycles of 95 °C for 10 s, 60 °C for 15 s. Fluorescence was recorded after each cycle, and melting curve analysis was performed from 65 °C to 95 °C. Reference genes were selected based on a low coefficient of variation of expression reported in literature and uniform expression for all cDNA samples for each of the above described experiments.

*VviERF6L1* overexpression was tested upon receival of transgenic plants with PCR of GFP and semi-quantitative PCR of *VviERF6L1*. GFP expression was confirmed with PCR (95 °C for 2 min, 35 cycles of 95 °C for 30 s, 58 °C for 30 s, 72 °C for 30 s) using GFP specific primers. Semi-quantitative PCR was performed on 1.0 μg of reverse transcribed RNA from two leaves of three individual plants per line with gene specific primers. The reaction consisted of 7 μL 10-fold diluted cDNA, 3.5 μL each 10 μM forward and reverse primers, 35 μL GoTaq Green Master Mix (Promega), and 21 μL DEPC water. Samples collected at cycles 23, 26, 29, 32, and 35 were run on 2% agarose gels stained with ethidium bromide to compare *VviERF6L1* amplification in overexpression lines relative to the empty vector control normalized to a ubiquitin reference gene. Several years after receival of the transgenic lines, stable overexpression was confirmed by RT-qPCR performed on 1.5 μg cDNA from three individual leaves harvested on 3 separate plants for each line with gene specific primers as before [122]. RT-qPCR was performed on cDNA samples reverse transcribed form 1.3 μg template RNA with iScript reverse transcriptase supermix (Bio-Rad) according to the manufacturer’s instructions using *VviGAPDH* and *VviACT7* as reference genes. RT-qPCR was conducted with SYBR Green Master Mix (Bio-Rad) for initial confirmation of overexpression lines and to confirm results on the same samples analyzed with RNA-Seq. All other RT-qPCR were performed with 2 μg of RNA reverse transcribed to cDNA with iScript Reverse Transcriptase Supermix (Bio-Rad) according to manufacturer’s instructions. RT-qPCR was conducted for overexpression confirmation, chilling, ABA, and respective control treatments with SYBR Green Master Mix (Bio-Rad). RT-qPCR was conducted to confirm *VviERF6L1* expression and for cold treatments and controls with GoTaq® qPCR Master Mix (Promega). Normalized relative quantity was calculated according to Hellemans et al. [101]. *VviACT7* and *VviGAPDH* were used as reference genes for the ABA treatment. *VviABF2* was used as a positive control gene for the ABA treatment. *VviUbi* and *VviACT7* were used as reference genes, and V*viCBF1* was used as a positive control gene.

### RNA-Seq analysis of *VviERF6L1* overexpression lines

Leaf RNA samples from three individual L12–3, L12–11, L12–23 and G1 vines were sequenced with Illumina TruSeq 2500 at the University of California, Los Angeles Genomic Center, to produce 36 bp single end reads. Each sample was sequenced on two different lanes (technical replicates). Read quality for each sample was verified with FastQC version 0.11.8 before and after trimming adaptors based on released adaptor sequences [117]. Sample 12–23-2_S8 had ~ 4.4% library size compared to the average library size of the other samples. Sample 12–23-2_S8 was excluded from further analysis. Over-represented sequences were extracted and identified with blast+ version 2.8.0 alpha [126]. Reads from both sequencing lanes were concatenated per sample. Transcript abundance was quantified with Salmon with standard settings for single end reads [119]. Tximport version 1.8.0 [127] followed by DESeq2 version 1.20.0 [120] were implemented to perform differential expression analysis. Venn diagrams were created in R with the package limma version 3.36.5 [117]. Heatmaps were created in R with ComplexHeatmap [128].

### Statistical analysis

Statistical analysis performed to compare multiple means included the student t-test, one- and two-way ANOVAs followed by Tukey’s HSD test after assumptions were met. Letters or asterisks indicate statistical significance between the specified comparisons. The error rate α = 0.05 was used in all comparisons. Statistical analyses were performed using R version 3.4.3.

## Supplementary information

**Additional file 1.** PN40024 VviERF6L protein motif logos. Protein motif logos of PN40024 VviERF6Ls determined by MEME. X-axis represents relative residue position in motif. Y-axis letter height (bits) indicates relative frequency of a residue at a given position in the motif across the VviERF6L proteins. Left side colors correspond to Fig. 1.

**Additional file 2.** PN40024 and Cabernet Sauvignon (CS) VviERF6L protein motif consensus sequences. Motif number (given based on E-value ranking), amino acid sequence, conservation (E-value), and length (amino acid residues) of the nine highly conserved protein motifs in PN40024 and CS VviERF6L proteins.

**Additional file 3.** PN40024 VviERF6L protein motif coordinates including consensus motif, and VviERF6L specific motif sequence, start and stop residue positions.

**Additional file 4. **PN40024 VviERF6L ERF domain percent identity with closest *Arabidopsis thaliana* ERF domain ortholog.

**Additional file 5. **The number of *ERF6L* paralogs across species. The number of *ERF6Ls* in species (carrot (*D. carota*), soybean (*G. max*), tomato (*S. lycopersicum*), and potato (*S. tuberosum*)) identified being closely related to *VviERF6L1* from the Pantaxonomic Compara Gene Tree on Gramene (2018 version containing 44 species) using the V3 annotation of PN40024.

**Additional file 6. **Cabernet Sauvignon (CS) VviERF6L protein motif presence and abundance. The frequency of the 13 highly conserved amino acid motifs (right) in the 26 translated CS *VviERF6L* genes (bottom). Exact motif coordinates are in Additional File 8.

**Additional file 7.** Cabernet Sauvignon (CS) VviERF6L protein motif logos. Protein motif logos of CS VviERF6Ls determined by MEME. X-axis represents relative residue position in motif. Y-axis letter height (bits) indicates relative frequency of a residue at a given position in the motif across the VviERF6L proteins. Left side colors corresponding to PN40024 motifs based on percent identity.

**Additional file 8.** Cabernet Sauvignon (CS) *VviERF6L* protein motif coordinates including start position, motif number and consensus motif.

**Additional file 9.** CS *VviERF6L* protein motifs. Relative position of protein motifs from N-terminus (bottom) to C-terminus (top) of CS *VviERF6Ls* determined by MEME. Motif number based on E-value and sequence from Additional File 2 represented by colors (upper right). For exact motif coordinates of each CS *VviERF6L* see Additional File 8. CS VviERF6L motifs with corresponding PN40024 motifs share colors with Fig. 1.

**Additional file 10.** Percent identity of PN40024 and Cabernet Sauvignon (CS) VviERF6L protein motifs.

**Additional file 11.** Cabernet Sauvignon (CS) *VviERF6L* gene names, protein sequences and protein length. Average length of all 26 at bottom.

**Additional file 12. **Maximum likelihood phylogenetic tree of PN40024 V2 assembly V3 structural annotation, CS, CH, and CA VviERF6L and all PN40024 VviERF proteins. Maximum likelihood method and Jones-Taylor-Thornton matrix-based model; Bootstrap consensus tree inferred from *n*=1,000 replicates. The percentage of bootstrap replicates in which associated proteins clustered together are shown as numbers next to the branches. The initial tree for the heuristic search generated from the maximum parsimony method. *n*= 217 amino acid sequences and 1886 positions in the final data set. Evolutionary analysis from MEGA X; Subtree-Pruning-Regrafting - Fast (SPR level 3).

**Additional file 13. **PN40024, CS, CA, and CH *VviERF6Ls* and all PN40024 VviERF protein sequences used to create phylogenetic tree.

**Additional file 14. **PN40224 *VviERF6L* putative promoter region (-3000 bp) motif coordinates. Motif name, start site relative to transcription start site, sequence, PLACE identification number, Vitis gene name, and corresponding *VviERF6L* name are given.

**Additional file 15. **PN40024 *VviERF6L* promoter (-3000 bp) motif frequency. Including motif name, frequency in each *VviERF6L* denoted by common and gene names.

**Additional file 16. **Number and location of PN40024 cis-regulatory elements most abundant in *VviERF6L12* relative to all other *VviERF6L* promoter regions. The ACGTATERD1 (red), LECPLEACS2 (green), SEF1MOTIF (blue), and WBOXATNPR1 (purple) were amongst the most abundant promoter motifs in *VviERF6L12*. Each occurrence of a motif is marked as a single hit at its appropriate position from the transcription start site (TSS) at position 0. Motif nucleotide sequence denoted in corresponding color to hits. Complete cisregulatory element data is located in Additional Files 14 and 15.

**Additional file 17. **Expression of *VviERF6Ls* across grapevine tissues and organs from the grapevine expression Atlas.

**Additional file 18. ***VviERF6L* gene expression in berry pulp, seed, and skin across berry development. Log2(RMA-normalized signal intensity+1) gene expression of 12 *VviERF6Ls* from Pinot Noir clone Pommard berry pulp (dark), seed (light), and skin (white) at pre-veraison (PRV), pink-soft (PS) berries at mid-ripening, and red-soft (RSH) berries at maturity [GSE49569]; mean ± SE.

**Additional file 19. **Meta-data analysis of microarrays and RNA-seq series downloaded from NCBI GEO and SRA and investigated for VviERF6L expression.

**Additional file 20. ** Annotation and number of cross hybridizing *VviERF6L* probes.

**Additional file 21. **RT-qPCR results of exogenous ABA application. Mature detached CS leaves were sprayed with exogenous 10 μM ABA (protone) or water control. Leaves were collected one hour after treatment. Control in pink and ABA treated in blue with bars as mean ± SE; *n* = 3.

**Additional file 22. **Differential expression analysis contrasts of interest for PRJNA516950. Including contrast number (arbitrarily assigned), species, treatment, organ, and week for both sample groups being compared.

**Additional file 23.** The number of differential expression analysis (DEA) contrasts of interest (COI) in which a *VviERF6L* was a differentially expressed gene (DEG) in PRJNA516950. *VviERF6Ls *are ordered from highest to lowest number of COI in which a *VviERF6L* is a DEG. COI are listed in Additional File 22.

**Additional file 24. ***VviERF6L* gene expression in response to chilling. MAS5-calculated signal intensity of *VviERF6Ls* in CS shoot tips of vines exposed to 22 °C control or 5 °C chilling treatment for 0, 4, and 8 hours [GSE31594]; mean ± SE.

**Additional file 25. ***VviERF6L1* did not respond to cold in Cabernet Sauvignon leaves. Expression of *CBF1 *(top) and *VviERF6L1* (bottom) in CS leaves after 2 hours of 4° C chilling treatment represented as NRQ measured with RT-qPCR, mean ± SE, *n* = 5 rounds of three individual leaves from individual plants. Control and chilling are represented as blue and pink respectively.

**Additional file 26. ***VviERF6L* gene expression in response to summer and winter harvest. Log_2_(FPKM+1) gene expression of 12 *VviERF6Ls* from berry pericarp of CS (light) and Riesling (dark) at three stages of ripening (EL35, 36, and 38) under a dual cropping system with harvesting in summer and winter [GSE103226]; mean ± SE.

**Additional file 27.** *VviERF6L* gene expression in response to *Erysiphe necator* infection. Log_2_(TPM+1) gene expression of 18 *VviERF6Ls* from leaves of *Vitis vinifera* cv. Carignan and Chinese *Vitis* accession DVIT3351.27 (DVIT3351), Husseine, Karadshandal, Khalchii, O34-16, Sochal, and Valilov mock (dark) or inoculated (light) with *Erysiphe necator* 1- and 5-days post infection (DPI) [GSE67191]; mean ± SE.

**Additional file 28. ***VviERF6L* gene expression in CS and SG pericarp over berry development across vineyards and years. Log_2_(RMA-normalized signal intensity+1) gene expression of 12 *VviERF6Ls* from Cabernet Sauvignon (CS (dark)) and Sangiovese (SG (light)) berry pericarp from three vineyards located in Bolgheri, Montalcino, and Riccone Italy in 2011 and 2012 over pea-size (PS), pre-veraison (PV), mid-ripening (MR), and fully ripened (FR) stages of development; mean ± SE.

**Additional file 29. ***VviERF6L* gene expression in Corvina pericarp over berry development across vineyards and years. Log_2_(RMA-normalized signal intensity+1) gene expression of 12 *VviERF6Ls* from Corvina pericarp from four representative Italian vineyards (abbreviated names from original paper; meaning nondisclosed) from 2006-2008 at veraison (V), mid-ripening (MR), and harvest (H) [GSE41633]; mean ± SE.

**Additional file 30.** Venn diagram of gene co-expression analysis. Co-expression analysis was performed on the 18-member *VviERF6L* clade in the five data series reanalyzed with the PN40024 V3 annotation. Number of genes sharing expression patterns for data series represented in cross-sections from top 100 co-expressed genes. Number at bottom indicates genes that did not share expression pattern with the *VviERF6L* clade.

**Additional file 31.** List of genes co-expressed with *VviERF6L* clade in four out of five data series. Gene ID and corresponding annotation. The four RNA-Seq data series the genes were co-expressed with the *VviERF6L* clade is listed.

**Additional file 32.** Fold increase of *VviERF6L12* expression relative to average expression of all other *VviERF6Ls*. Expression value of *VviERF6L12* and average value of all other *VviERF6Ls* across all conditions for each data series with each data series' respective units. Fold increase of *VviERF6L12* expression taken as ratio of *VviERF6L12* expression relative to average expression value of all other *VviERF6Ls* across all conditions per data series.

**Additional file 33.** Verification of *VviERF6L1 *overexpression lines. (A) Semi-quant RT-qPCR of *GFP*, *VviERF6L1*, and *VviUbi1* from leaves at cycle 32. (B) Verification of *VviERF6L1* overexpression with RT-qPCR with *VviGAPDH *and *VviACT7* reference genes from individual leaves represented as a normalized relative quantity, mean ± SE, n = 3 individual leaves from 3 individual plants. Stars indicate significance between G1 (empty vector control) and *VviERF6L1* overexpression lines (*p*-value < 0.05) using student’s T-test. Blue and green corresponding to VviERF6L1 overexpression lines and empty vector control, respectively.

**Additional file 34.** Morphological phenotyping measurements taken that were not statistically significant between OX VviERF6L1 and empty vector control lines. Measurements were collected over the span of three weeks to 3 years. Data were tested for significant differences between empty vector control line (G1) and overexpression lines (L12-1, 2, 3, 11, and 23). Assumptions were met for tests used to determine significance.

**Additional file 35.** *VviERF6L* expression in *VviERF6L1* overexpression lines. For each overexpression line (L12-3, L12-11, L12-23) and the empty vector control (G1), an average TMP value was calculated and log_2_ transformed and colored from yellow (low value) to purple (high value) for each of the 18 *VviERF6Ls*, *n*= 3.

**Additional file 36.** Annotations of differentially expressed genes in three *VviERF6L1* overexpression lines relative to empty vector control.

**Additional file 37. **Venn Diagram of differentially expressed genes between L12-3, -11, and -23 *VviERF6L1* overexpression lines relative to G1 empty vector control. Number of upregulated genes presented in black and down regulated genes presented as grey.

**Additional file 38.** Primers used for RT-qPCR analysis. Primers were designed using primer 3 and NCBI's primer design tool. Primers were designed to be specific for genes of interest and respectful of RT-qPCR settings and specifications.

## Data Availability

RNA-Seq data from the *VviERF6L1* overexpression and empty vector control lines were deposited in the Sequence Read Archive (SRA) database with the accession number PRJNA605564.

## Abbreviations

% ID: Percent identity
A + F: Acclimation with freezing
AA: Amino acid
ACC: Chilling acclimation
ACT7: Actin 7
At: *Arabidopsis thaliana*
bp: Base pair
C: Control
CA: *Vitis vinifera* (L.) cv. Carménère
CBF1: C-repeat/DRE binding factor 1
CDS: Coding sequence
CS: *Vitis vinifera* (L.) cv. Cabernet Sauvignon clone 8
CH: *Vitis vinifera* (L.) cv. Chardonnay
COI: Contrasts of interest
DEA: Differential expression analysis
DEG: Differentially expressed gene
DEPC: Diethyl pyrocarbonate
DI: De-ionized
ERF: Ethylene Response Factor
fdr: False discovery rate
FR: Fully ripened
FRZ: Freezing
GAPDH: Glyceraldehyde-3-Phosphate Dehydrogenase of Plastid
GEO: NCBI Gene Expression Omnibus
GFP: Green fluorescent protein
GH: Green-hard
GS: Green-soft
GSH: Green-soft at harvest
H: Harvest
Harv: Harvest
IW: Inoculated-wounded
JA: Jasmonic acid
LAR: Leucoanthocyanidin reductase
MEME: Multiple Em for Motif Elicitation
min: Minute
MR: Mid-ripening
NCBI: National Center for Biotechnology Information
NINW: Non-inoculated - non-wounded
NIW: Non-inoculated - wounded
NRQ: Normalized relative quantity
OX: Overexpression
Pea: The stage of berry development in which berries are the size of peas
Pro: 3000 base pairs from transcription start site
PRV: Pre-veraison
PS: Pea-sized
PSH: Pink-soft at harvest
PV: Post-veraison
RA: *Vitis champinii* (Ramsey)
RI: *Vitis riparia* (Riparia)
RNA-Seq: Ribo-nucleic acid-sequencing
RS: Red-soft
RSH: Red-soft at harvest
RT-qPCR: Reverse transcription-quantitative polymerase chain reaction
s: Second
SC: *Vitis vinifera x Vitis girdiana* (SC2)
SG: Sangiovese
Soft: The stage of berry development in which berries begin to soften
SRA: NCBI Sequence Read Archive
Touch: The stage of berry development in which berries begin to touch each other
TPM: Transcripts per million
TSS: Transcription start site
*VviUbi1*: *Vitis vinifera* ubiquitin 1
W: Week
WD: Water deficit
WT: Wild type
V: Veraison
Vs.: Versus
VviERF6L1: *Vitis vinifera* Ethylene Response Factor 6-Like 1
