# Supplementary material for: VviERF6Ls: an expanded clade in Vitis responds transcriptionally to abiotic and biotic stresses and berry development
Source: BMC Genomics. 2020 Jul 9;21:472. doi: 10.1186/s12864-020-06811-8 (PMC7350745; doi:10.1186/s12864-020-06811-8)
Supplement: Supplementary file 1 — Additional file 1. PN40024 VviERF6L protein motif logos. Protein motif logos of PN40024 VviERF6Ls determined by MEME. X-axis represents relative residue position in motif. Y-axis letter height (bits) indicates relative frequency of a residue at a given position in the motif across the VviERF6L proteins. Left side colors correspond to Fig. 1. [file 12864_2020_6811_MOESM1_ESM.pdf]

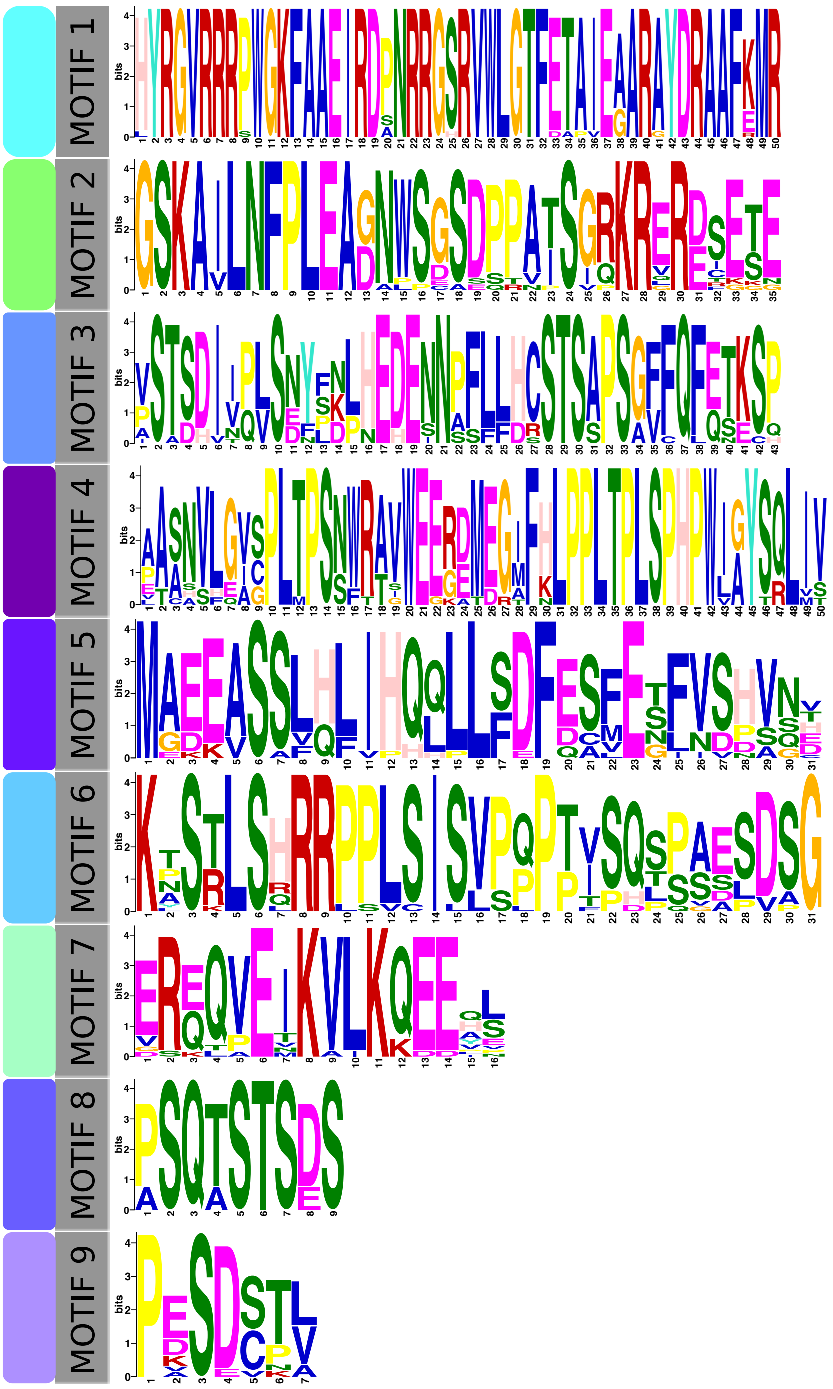


**Additional File 1: PN40024 VviERF6L protein motif logos.** Protein motif logos of PN40024 VviERF6Ls determined by MEME. X-axis represents relative residue position in motif. Y-axis letter height (bits) indicates relative frequency of a residue at a given position in the motif across the VviERF6L proteins. Left side colors correspond to Figure 1.
