# Supplementary material for: VviERF6Ls: an expanded clade in Vitis responds transcriptionally to abiotic and biotic stresses and berry development
Source: BMC Genomics. 2020 Jul 9;21:472. doi: 10.1186/s12864-020-06811-8 (PMC7350745; doi:10.1186/s12864-020-06811-8)
Supplement: Supplementary file 5 — Additional file 5. The number of ERF6L paralogs across species. The number of ERF6Ls in species (carrot (D. carota), soybean (G. max), tomato (S. lycopersicum), and potato (S. tuberosum)) identified being closely related to VviERF6L1 from the Pantaxonomic Compara Gene Tree on Gramene (2018 version containing 44 species) using the V3 annotation of PN40024. [file 12864_2020_6811_MOESM5_ESM.pdf]

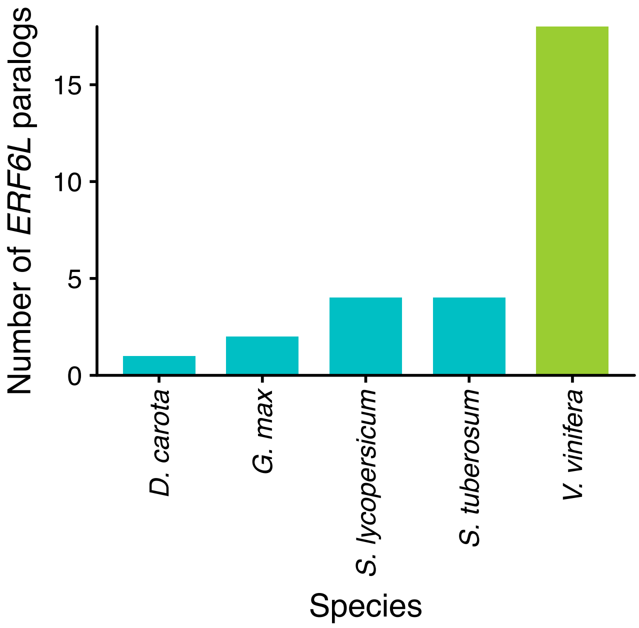

Additional File 5: The number of *ERF6L* paralogs across species. The number of *ERF6Ls* in species (carrot (*D. carota*), soybean (*G. max*), tomato (*S. lycopersicum*), and potato (*S. tuberosum*)) identified being closely related to *VviERF6L1* from the Pan-taxonomic Compara Gene Tree on Gramene (2018 version containing 44 species) using the V3 annotation of PN40024.
