# Supplementary material for: VviERF6Ls: an expanded clade in Vitis responds transcriptionally to abiotic and biotic stresses and berry development
Source: BMC Genomics. 2020 Jul 9;21:472. doi: 10.1186/s12864-020-06811-8 (PMC7350745; doi:10.1186/s12864-020-06811-8)
Supplement: Supplementary file 6 — Additional file 6. Cabernet Sauvignon (CS) VviERF6L protein motif presence and abundance. The frequency of the 13 highly conserved amino acid motifs (right) in the 26 translated CS VviERF6L genes (bottom). Exact motif coordinates are in Additional File 8. [file 12864_2020_6811_MOESM6_ESM.pdf]

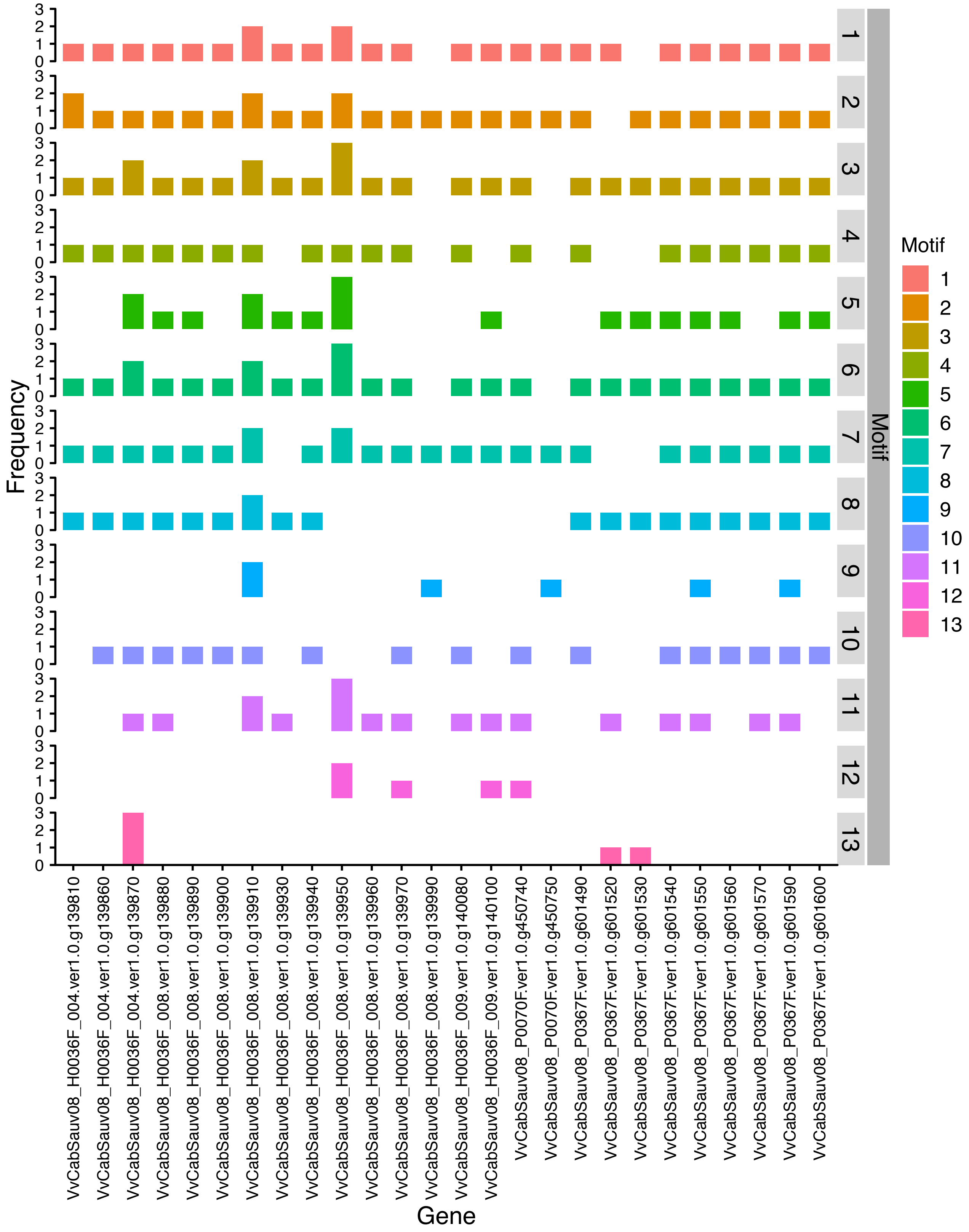

Additional File 6: Cabernet Sauvignon (CS) VviERF6L protein motif presence and abundance. The frequency of the 13 highly conserved amino acid motifs (right) in the 26 translated CS *VviERF6L* genes (bottom). Exact motif coordinates are in Additional File 8.
