# Supplementary material for: VviERF6Ls: an expanded clade in Vitis responds transcriptionally to abiotic and biotic stresses and berry development
Source: BMC Genomics. 2020 Jul 9;21:472. doi: 10.1186/s12864-020-06811-8 (PMC7350745; doi:10.1186/s12864-020-06811-8)
Supplement: Supplementary file 7 — Additional file 7. Cabernet Sauvignon (CS) VviERF6L protein motif logos. Protein motif logos of CS VviERF6Ls determined by MEME. X-axis represents relative residue position in motif. Y-axis letter height (bits) indicates relative frequency of a residue at a given position in the motif across the VviERF6L proteins. Left side colors corresponding to PN40024 motifs based on percent identity. [file 12864_2020_6811_MOESM7_ESM.pdf]

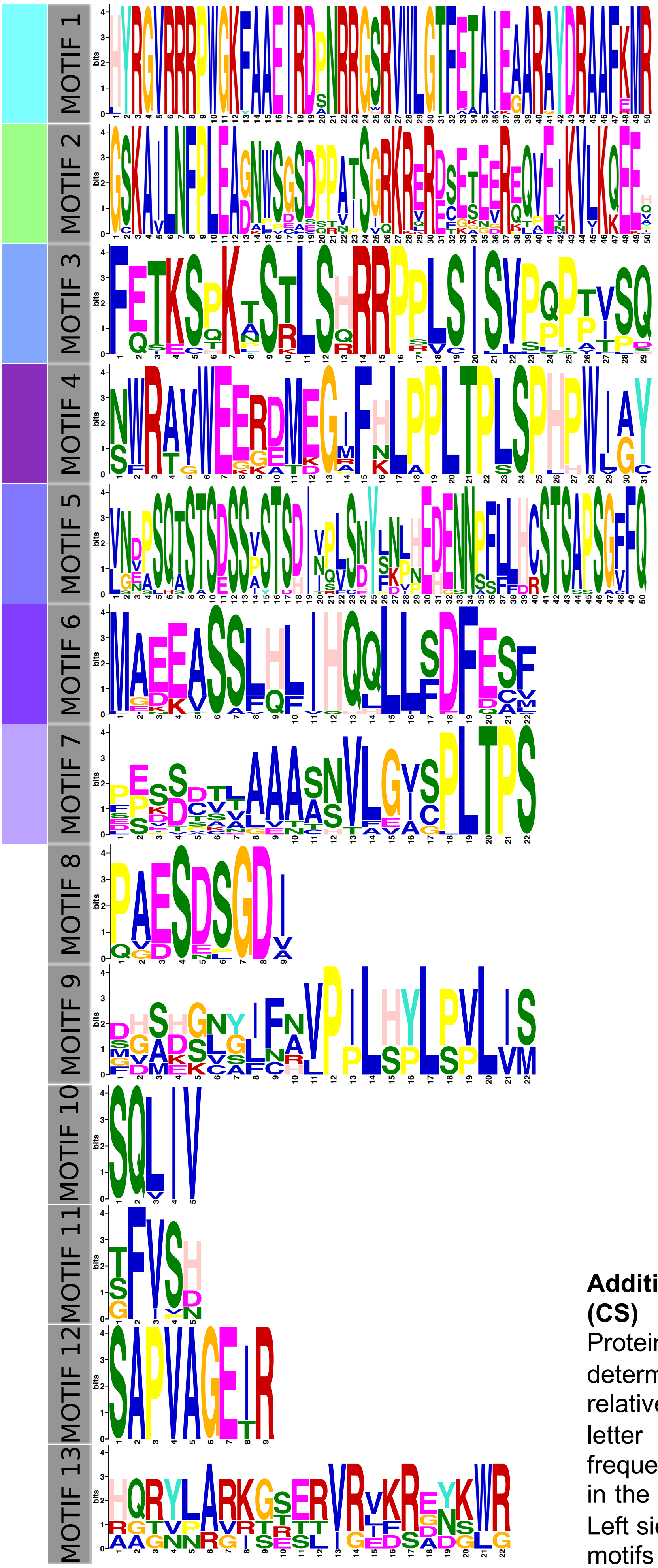

Additional File 7: Cabernet Sauvignon (CS) VviERF6L protein motif logos. Protein motif logos of CS VviERF6Ls determined by MEME. X-axis represents relative residue position in motif. Y-axis letter height (bits) indicates relative frequency of a residue at a given position in the motif across the VviERF6L proteins. Left side colors corresponding to PN40024 motifs based on percent identity.
