# Supplementary figures and images for: VviERF6Ls: an expanded clade in Vitis responds transcriptionally to abiotic and biotic stresses and berry development
Source: BMC Genomics. 2020 Jul 9;21:472. doi: 10.1186/s12864-020-06811-8 (PMC7350745; doi:10.1186/s12864-020-06811-8)

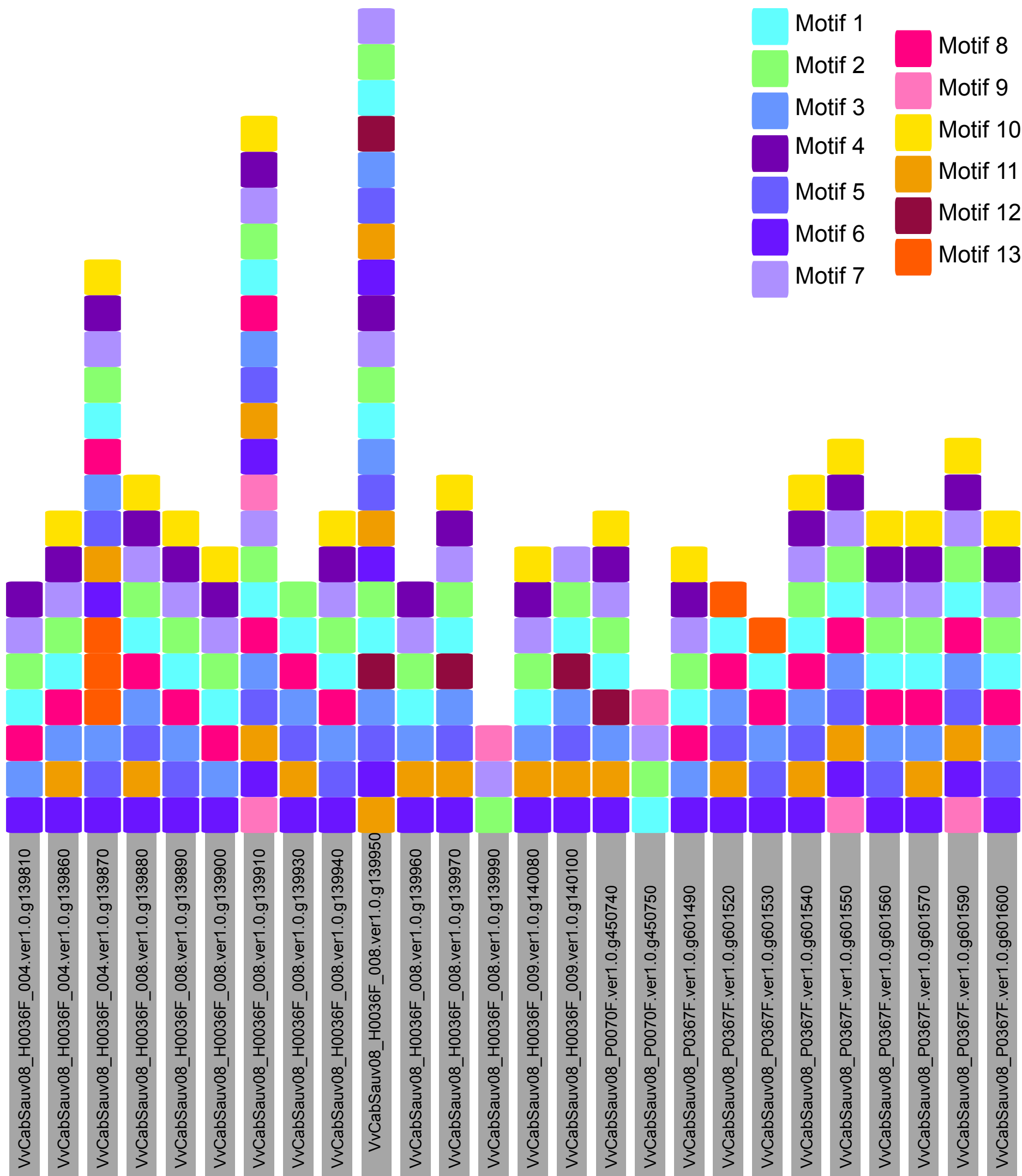

Supplement: Supplementary file 9 — Additional file 9. CS VviERF6L protein motifs. Relative position of protein motifs from N-terminus (bottom) to C-terminus (top) of CS VviERF6Ls determined by MEME. Motif number based on E-value and sequence from Additional File 2 represented by colors (upper right). For exact motif coordinates of each CS VviERF6L see Additional File 8. CS VviERF6L motifs with corresponding PN40024 motifs share colors with Fig. 1. [file 12864_2020_6811_MOESM9_ESM.pdf]

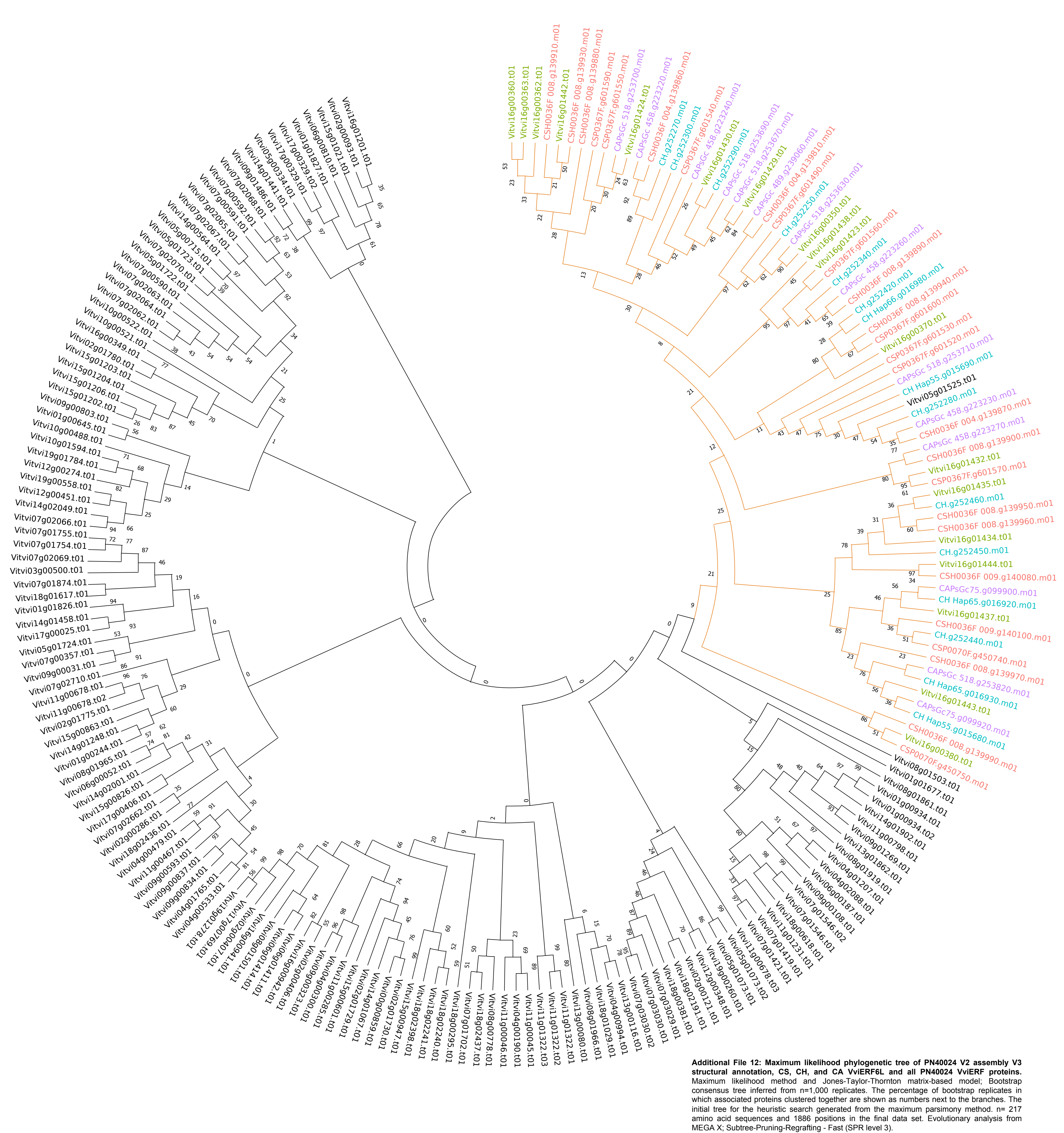

Supplement: Supplementary file 12 — Additional file 12. Maximum likelihood phylogenetic tree of PN40024 V2 assembly V3 structural annotation, CS, CH, and CA VviERF6L and all PN40024 VviERF proteins. Maximum likelihood method and Jones-Taylor-Thornton matrix-based model; Bootstrap consensus tree inferred from n=1,000 replicates. The percentage of bootstrap replicates in which associated proteins clustered together are shown as numbers next to the branches. The initial tree for the heuristic search generated from the maximum parsimony method. n= 217 amino acid sequences and 1886 positions in the final data set. Evolutionary analysis from MEGA X; Subtree-Pruning-Regrafting - Fast (SPR level 3). [file 12864_2020_6811_MOESM12_ESM.pdf]
