# Supplementary material for: VviERF6Ls: an expanded clade in Vitis responds transcriptionally to abiotic and biotic stresses and berry development
Source: BMC Genomics. 2020 Jul 9;21:472. doi: 10.1186/s12864-020-06811-8 (PMC7350745; doi:10.1186/s12864-020-06811-8)
Supplement: Supplementary file 16 — Additional file 16. Number and location of PN40024 cis-regulatory elements most abundant in VviERF6L12 relative to all other VviERF6L promoter regions. The ACGTATERD1 (red), LECPLEACS2 (green), SEF1MOTIF (blue), and WBOXATNPR1 (purple) were amongst the most abundant promoter motifs in VviERF6L12. Each occurrence of a motif is marked as a single hit at its appropriate position from the transcription start site (TSS) at position 0. Motif nucleotide sequence denoted in corresponding color to hits. Complete cisregulatory element data is located in Additional Files 14 and 15. [file 12864_2020_6811_MOESM16_ESM.pdf]

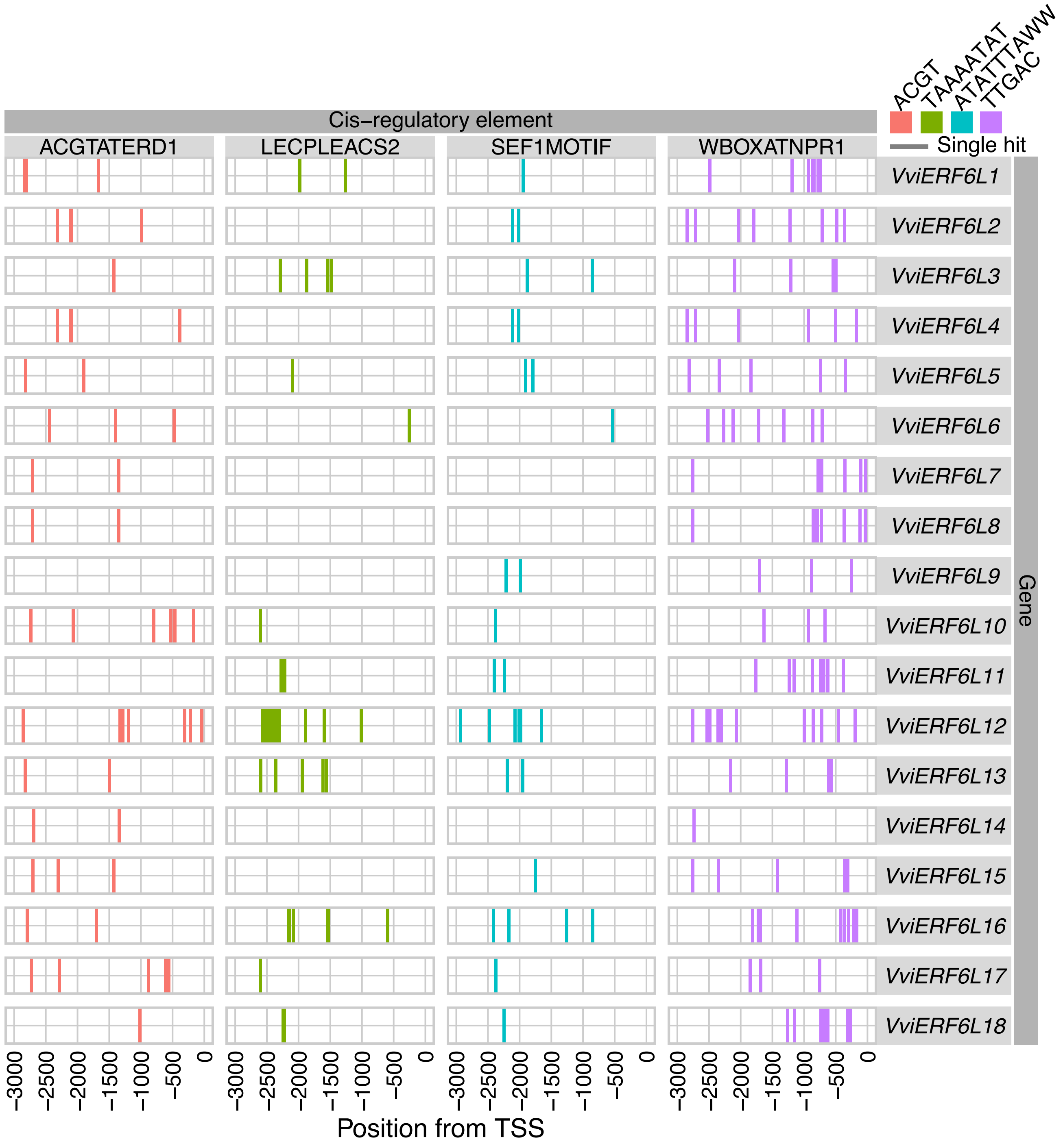

Additional File 16: Number and location of PN40024 cis-regulatory elements most abundant in *VviERF6L12* relative to all other *VviERF6L* promoter regions. The ACGTATERD1 (red), LECPLEACS2 (green), SEF1MOTIF (blue), and WBOXATNPR1 (purple) were amongst the most abundant promoter motifs in *VviERF6L12*. Each occurrence of a motif is marked as a single hit at its appropriate position from the transcription start site (TSS) at position 0. Motif nucleotide sequence denoted in corresponding color to hits. Complete cis-regulatory element data is located in Additional Files 14 and 15.
