# Supplementary material for: VviERF6Ls: an expanded clade in Vitis responds transcriptionally to abiotic and biotic stresses and berry development
Source: BMC Genomics. 2020 Jul 9;21:472. doi: 10.1186/s12864-020-06811-8 (PMC7350745; doi:10.1186/s12864-020-06811-8)
Supplement: Supplementary file 18 — Additional file 18. VviERF6L gene expression in berry pulp, seed, and skin across berry development. Log2(RMA-normalized signal intensity+1) gene expression of 12 VviERF6Ls from Pinot Noir clone Pommard berry pulp (dark), seed (light), and skin (white) at pre-veraison (PRV), pink-soft (PS) berries at mid-ripening, and red-soft (RSH) berries at maturity [GSE49569]; mean ± SE. [file 12864_2020_6811_MOESM18_ESM.pdf]

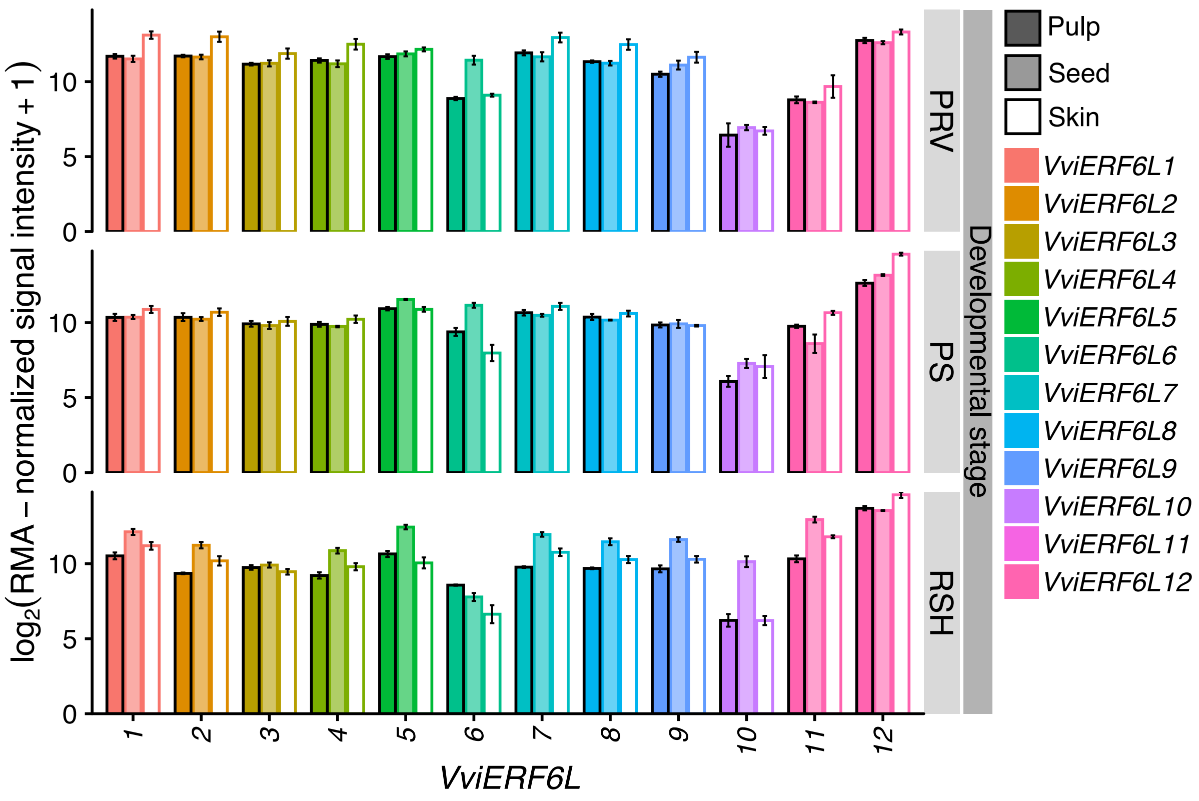


**Additional File 18: *VviERF6L* gene expression in berry pulp, seed, and skin across berry development.** $Log_2$(RMA-normalized signal intensity+1) gene expression of 12 *VviERF6Ls* from Pinot Noir clone Pommard berry pulp (dark), seed (light), and skin (white) at pre-veraison (PRV), pink-soft (PS) berries at mid-ripening, and red-soft (RSH) berries at maturity [GSE49569]; mean ± SE.
