# Supplementary material for: VviERF6Ls: an expanded clade in Vitis responds transcriptionally to abiotic and biotic stresses and berry development
Source: BMC Genomics. 2020 Jul 9;21:472. doi: 10.1186/s12864-020-06811-8 (PMC7350745; doi:10.1186/s12864-020-06811-8)
Supplement: Supplementary file 21 — Additional file 21. RT-qPCR results of exogenous ABA application. Mature detached CS leaves were sprayed with exogenous 10 μM ABA (protone) or water control. Leaves were collected one hour after treatment. Control in pink and ABA treated in blue with bars as mean ± SE; n = 3. [file 12864_2020_6811_MOESM21_ESM.pdf]

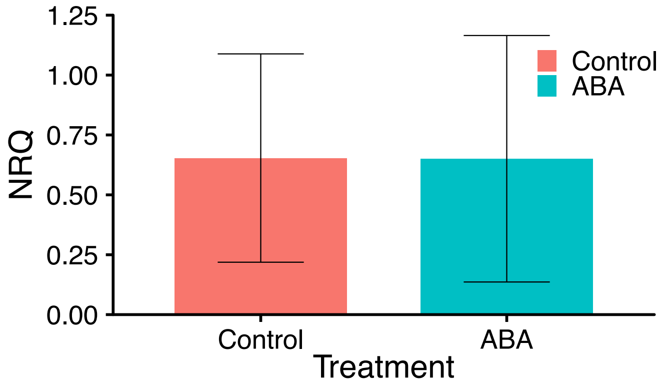

Additional File 21: RT-qPCR results of exogenous ABA application. Mature detached CS leaves were sprayed with exogenous 10 μM ABA (protone) or water control. Leaves were collected one hour after treatment. Control in pink and ABA treated in blue with bars as mean ± SE; n = 3.
