# Supplementary material for: VviERF6Ls: an expanded clade in Vitis responds transcriptionally to abiotic and biotic stresses and berry development
Source: BMC Genomics. 2020 Jul 9;21:472. doi: 10.1186/s12864-020-06811-8 (PMC7350745; doi:10.1186/s12864-020-06811-8)
Supplement: Supplementary file 23 — Additional file 23. The number of differential expression analysis (DEA) contrasts of interest (COI) in which a VviERF6L was a differentially expressed gene (DEG) in PRJNA516950. VviERF6Ls are ordered from highest to lowest number of COI in which a VviERF6L is a DEG. COI are listed in Additional File 22. [file 12864_2020_6811_MOESM23_ESM.pdf]

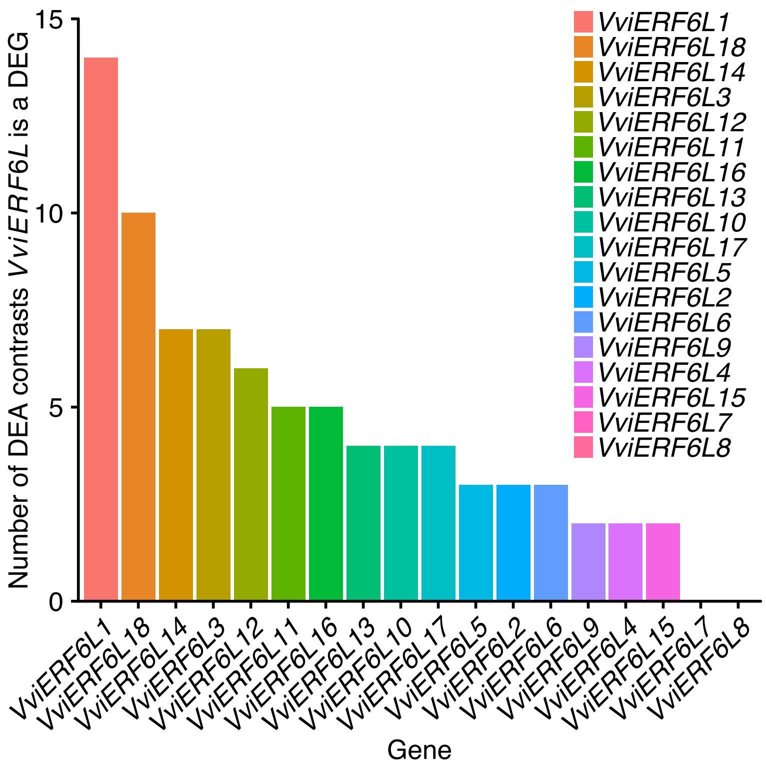

Additional File 23: The number of differential expression analysis (DEA) contrasts of interest (COI) in which a *VviERF6L* was a differentially expressed gene (DEG) in PRJNA516950. *VviERF6Ls* are ordered from highest to lowest number of COI in which a *VviERF6L* is a DEG. COI are listed in Additional File 22.
