# Supplementary material for: VviERF6Ls: an expanded clade in Vitis responds transcriptionally to abiotic and biotic stresses and berry development
Source: BMC Genomics. 2020 Jul 9;21:472. doi: 10.1186/s12864-020-06811-8 (PMC7350745; doi:10.1186/s12864-020-06811-8)
Supplement: Supplementary file 24 — Additional file 24. VviERF6L gene expression in response to chilling. MAS5-calculated signal intensity of VviERF6Ls in CS shoot tips of vines exposed to 22 °C control or 5 °C chilling treatment for 0, 4, and 8 hours [GSE31594]; mean ± SE. [file 12864_2020_6811_MOESM24_ESM.pdf]

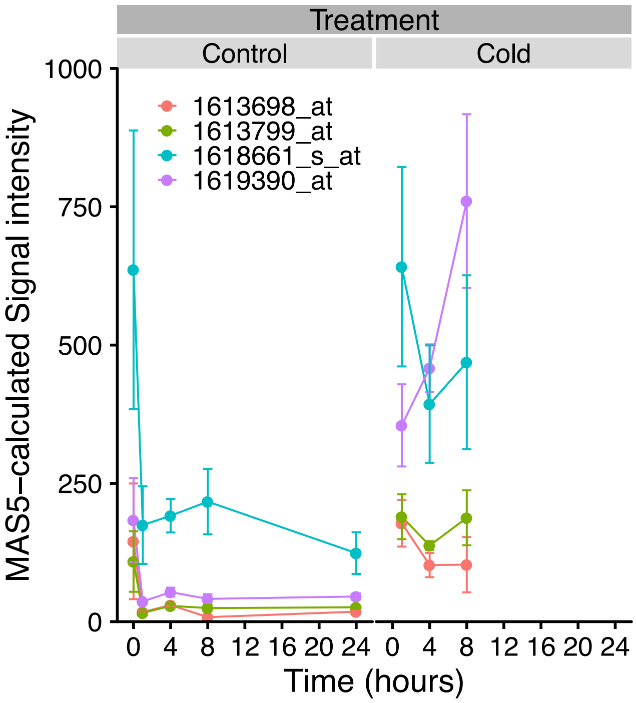

Additional File 24: *VviERF6L* gene expression in response to chilling. MAS5-calculated signal intensity of *VviERF6Ls* in CS shoot tips of vines exposed to 22 °C control or 5 °C chilling treatment for 0, 4, and 8 hours [GSE31594]; mean ± SE.
