# Supplementary material for: VviERF6Ls: an expanded clade in Vitis responds transcriptionally to abiotic and biotic stresses and berry development
Source: BMC Genomics. 2020 Jul 9;21:472. doi: 10.1186/s12864-020-06811-8 (PMC7350745; doi:10.1186/s12864-020-06811-8)
Supplement: Supplementary file 25 — Additional file 25. VviERF6L1 did not respond to cold in Cabernet Sauvignon leaves. Expression of CBF1 (top) and VviERF6L1 (bottom) in CS leaves after 2 hours of 4° C chilling treatment represented as NRQ measured with RT-qPCR, mean ± SE, n = 5 rounds of three individual leaves from individual plants. Control and chilling are represented as blue and pink respectively. [file 12864_2020_6811_MOESM25_ESM.pdf]

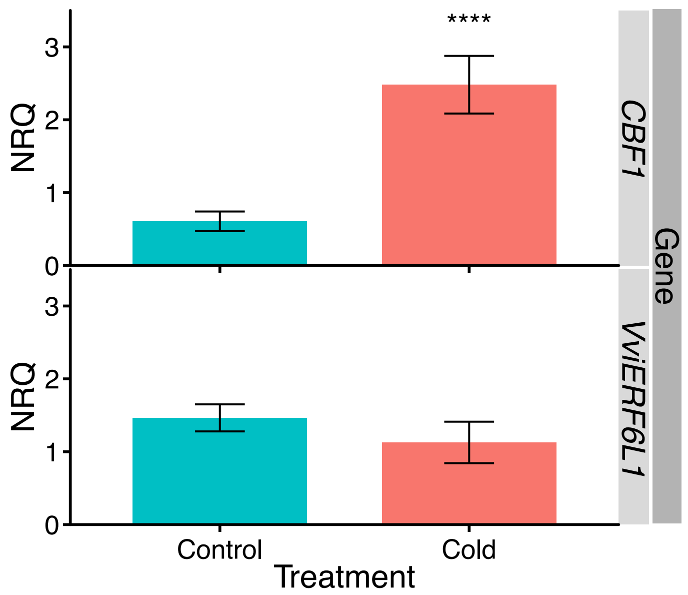

Additional File 25: *VviERF6L1* did not respond to cold in Cabernet Sauvignon leaves. Expression of *CBF1* (top) and *VviERF6L1* (bottom) in CS leaves after 2 hours of 4° C chilling treatment represented as NRQ measured with RT-qPCR, mean ± SE, n = 5 rounds of three individual leaves from individual plants. Control and chilling are represented as blue and pink respectively.
