# Supplementary material for: VviERF6Ls: an expanded clade in Vitis responds transcriptionally to abiotic and biotic stresses and berry development
Source: BMC Genomics. 2020 Jul 9;21:472. doi: 10.1186/s12864-020-06811-8 (PMC7350745; doi:10.1186/s12864-020-06811-8)
Supplement: Supplementary file 26 — Additional file 26. VviERF6L gene expression in response to summer and winter harvest. Log2(FPKM+1) gene expression of 12 VviERF6Ls from berry pericarp of CS (light) and Riesling (dark) at three stages of ripening (EL35, 36, and 38) under a dual cropping system with harvesting in summer and winter [GSE103226]; mean ± SE. [file 12864_2020_6811_MOESM26_ESM.pdf]

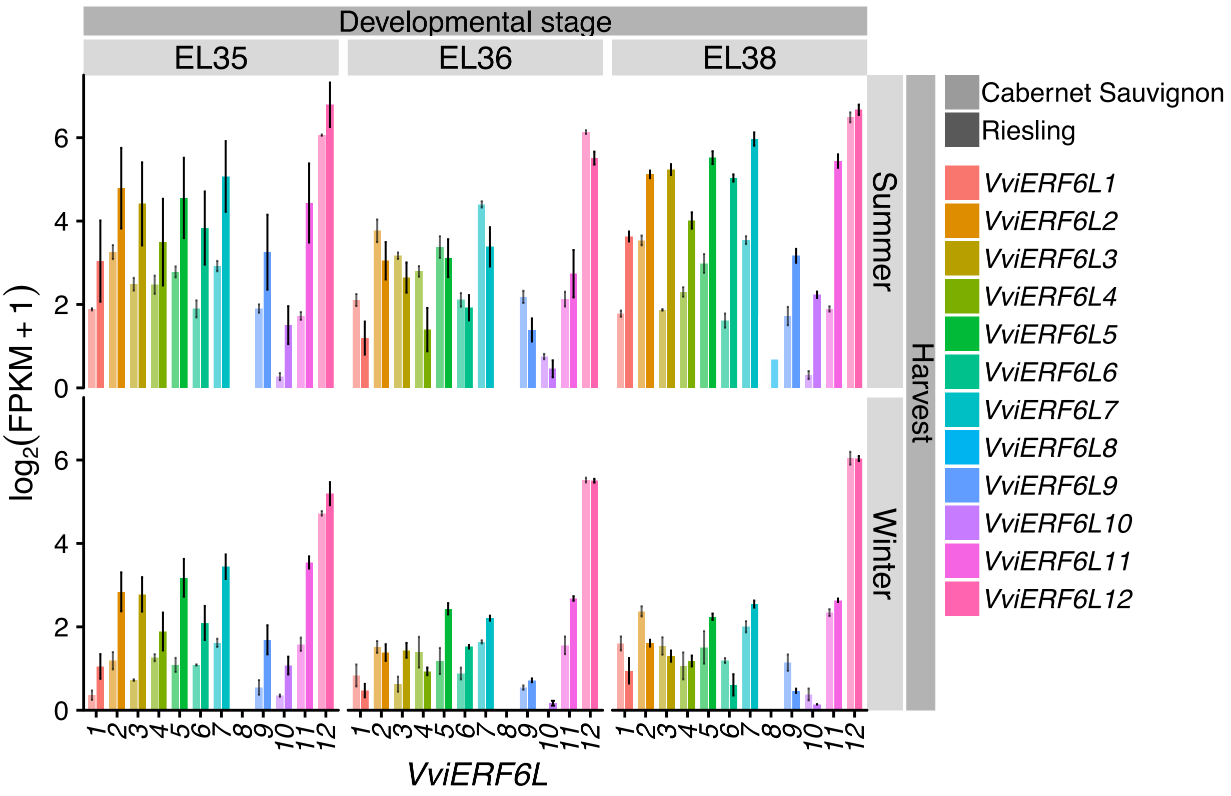

Additional File 26: *VviERF6L* gene expression in response to summer and winter harvest. Log_2(FPKM+1) gene expression of 12 *VviERF6Ls* from berry pericarp of CS (light) and Riesling (dark) at three stages of ripening (EL35, 36, and 38) under a dual cropping system with harvesting in summer and winter [GSE103226]; mean ± SE.
