# Supplementary material for: VviERF6Ls: an expanded clade in Vitis responds transcriptionally to abiotic and biotic stresses and berry development
Source: BMC Genomics. 2020 Jul 9;21:472. doi: 10.1186/s12864-020-06811-8 (PMC7350745; doi:10.1186/s12864-020-06811-8)
Supplement: Supplementary file 27 — Additional file 27. VviERF6L gene expression in response to Erysiphe necator infection. Log2(TPM+1) gene expression of 18 VviERF6Ls from leaves of Vitis vinifera cv. Carignan and Chinese Vitis accession DVIT3351.27 (DVIT3351), Husseine, Karadshandal, Khalchii, O34-16, Sochal, and Valilov mock (dark) or inoculated (light) with Erysiphe necator 1- and 5-days post infection (DPI) [GSE67191]; mean ± SE. [file 12864_2020_6811_MOESM27_ESM.pdf]

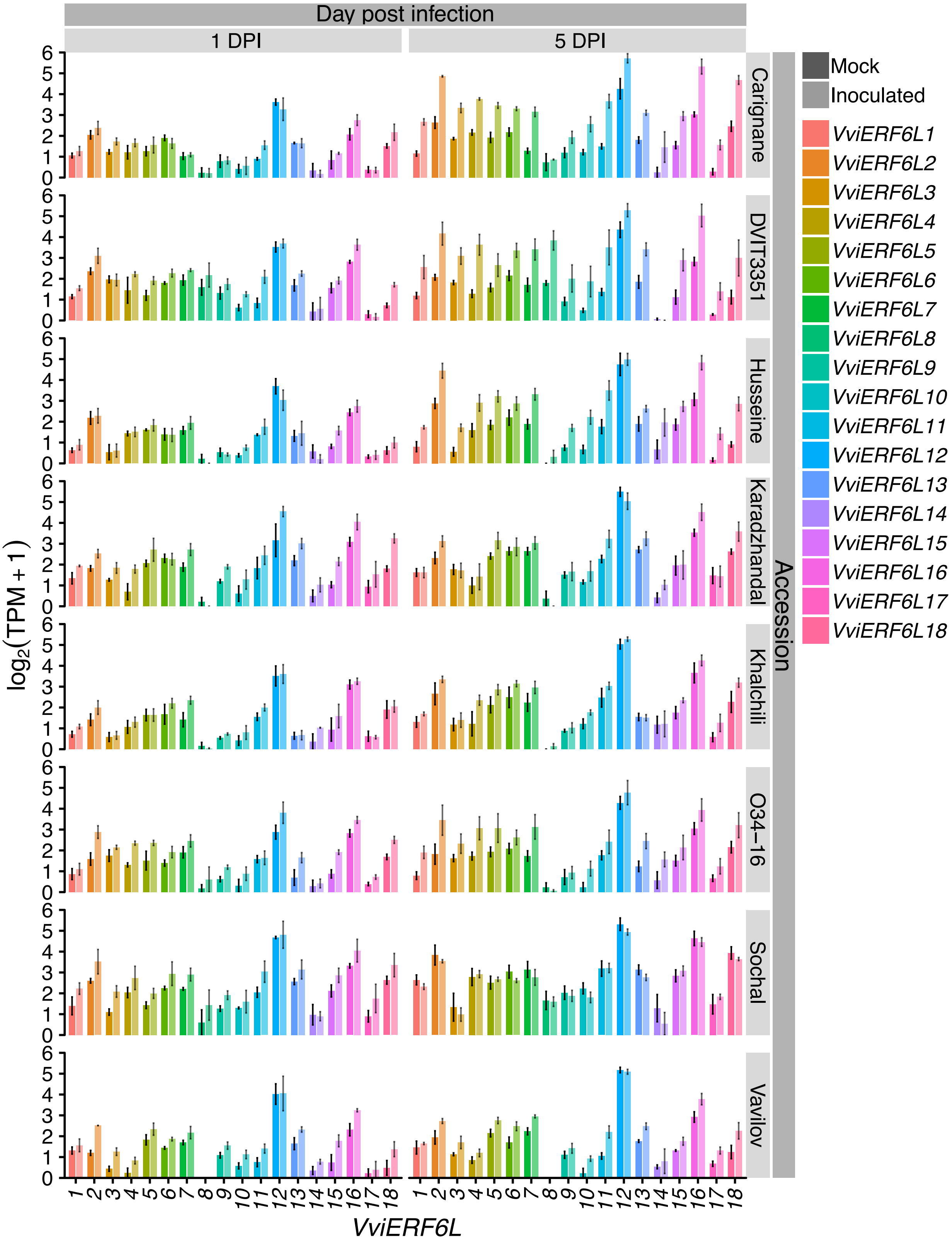

Additional File 27: *VviERF6L* gene expression in response to *Erysiphe necator* infection. Log_2(TPM+1) gene expression of 18 *VviERF6Ls* from leaves of *Vitis vinifera* cv. Carignan and Chinese *Vitis* accession DVIT3351.27 (DVIT3351), Husseine, Karadshandal, Khalchii, O34-16, Sochal, and Valilov mock (dark) or inoculated (light) with *Erysiphe necator* 1- and 5-days post infection (DPI) [GSE67191]; mean ± SE.
