# Supplementary material for: VviERF6Ls: an expanded clade in Vitis responds transcriptionally to abiotic and biotic stresses and berry development
Source: BMC Genomics. 2020 Jul 9;21:472. doi: 10.1186/s12864-020-06811-8 (PMC7350745; doi:10.1186/s12864-020-06811-8)
Supplement: Supplementary file 28 — Additional file 28. VviERF6L gene expression in CS and SG pericarp over berry development across vineyards and years. Log2(RMA-normalized signal intensity+1) gene expression of 12 VviERF6Ls from Cabernet Sauvignon (CS (dark)) and Sangiovese (SG (light)) berry pericarp from three vineyards located in Bolgheri, Montalcino, and Riccone Italy in 2011 and 2012 over pea-size (PS), pre-veraison (PV), mid-ripening (MR), and fully ripened (FR) stages of development; mean ± SE. [file 12864_2020_6811_MOESM28_ESM.pdf]

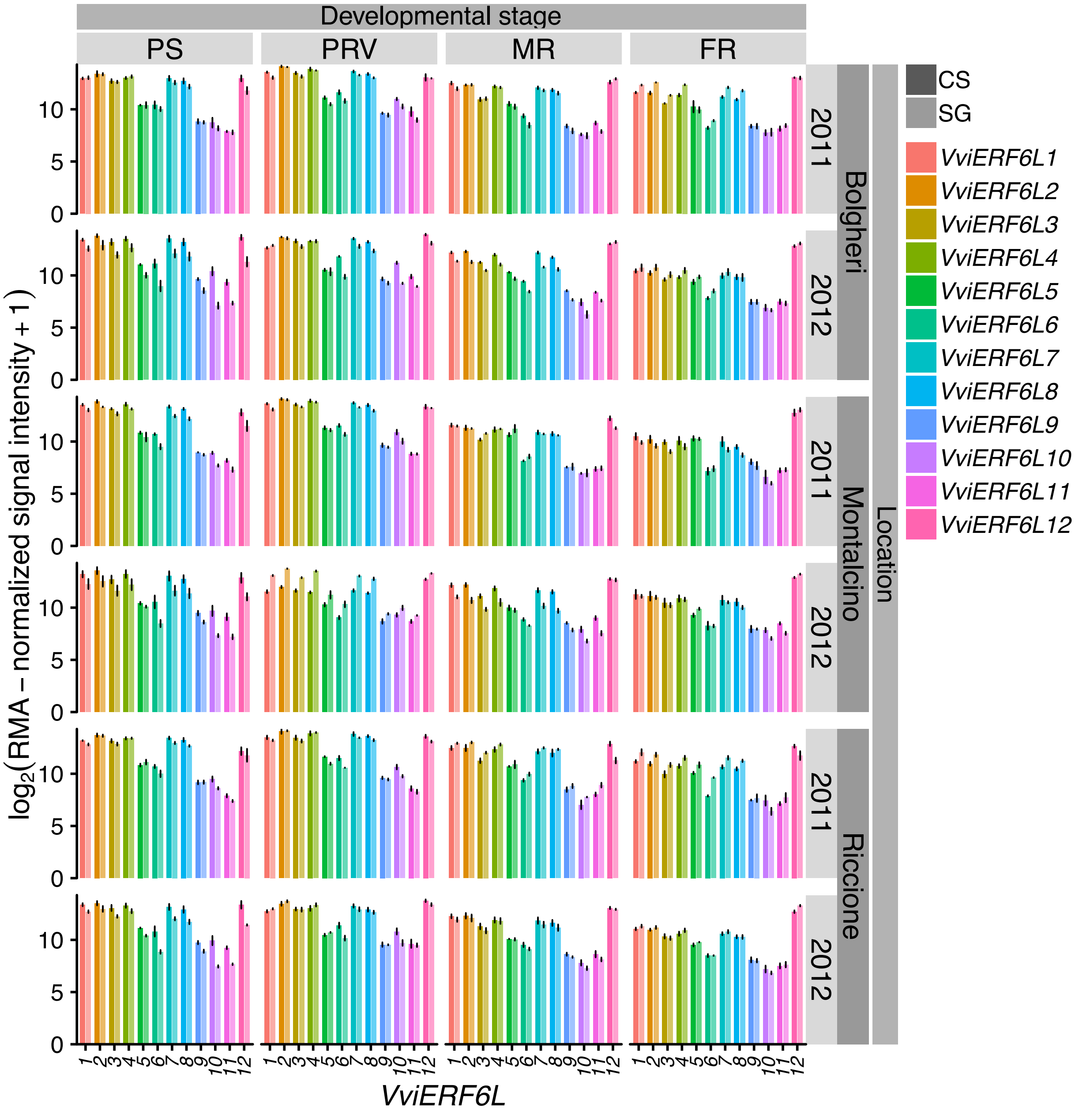

Additional File 28: *VviERF6L* gene expression in CS and SG pericarp over berry development across vineyards and years. Log_2(RMA-normalized signal intensity+1) gene expression of 12 *VviERF6Ls* from Cabernet Sauvignon (CS (dark)) and Sangiovese (SG (light)) berry pericarp from three vineyards located in Bolgheri, Montalcino, and Riccone Italy in 2011 and 2012 over pea-size (PS), pre-veraison (PV), mid-ripening (MR), and fully ripened (FR) stages of development; mean ± SE.
