# Supplementary material for: VviERF6Ls: an expanded clade in Vitis responds transcriptionally to abiotic and biotic stresses and berry development
Source: BMC Genomics. 2020 Jul 9;21:472. doi: 10.1186/s12864-020-06811-8 (PMC7350745; doi:10.1186/s12864-020-06811-8)
Supplement: Supplementary file 30 — Additional file 30. Venn diagram of gene co-expression analysis. Co-expression analysis was performed on the 18-member VviERF6L clade in the five data series reanalyzed with the PN40024 V3 annotation. Number of genes sharing expression patterns for data series represented in cross-sections from top 100 co-expressed genes. Number at bottom indicates genes that did not share expression pattern with the VviERF6L clade. [file 12864_2020_6811_MOESM30_ESM.pdf]

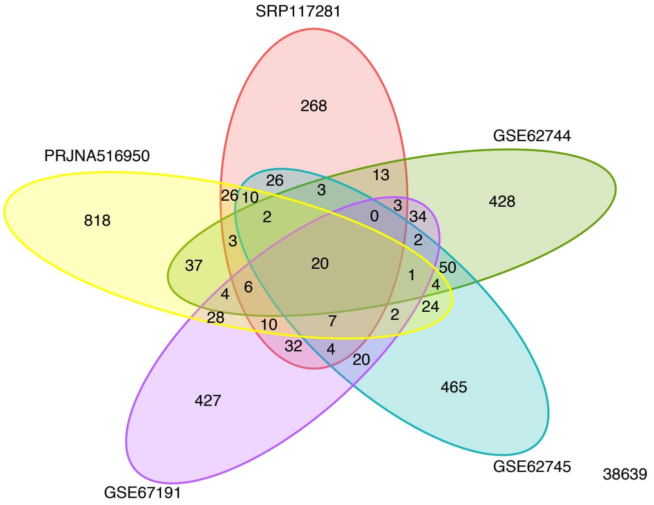

Additional File 30: Venn diagram of gene co-expression analysis. Co-expression analysis was performed on the 18-member *VviERF6L* clade in the five data series re-analyzed with the PN40024 V3 annotation. Number of genes sharing expression patterns for data series represented in cross-sections from top 100 co-expressed genes. Number at bottom indicates genes that did not share expression pattern with the *VviERF6L* clade
