# Supplementary material for: VviERF6Ls: an expanded clade in Vitis responds transcriptionally to abiotic and biotic stresses and berry development
Source: BMC Genomics. 2020 Jul 9;21:472. doi: 10.1186/s12864-020-06811-8 (PMC7350745; doi:10.1186/s12864-020-06811-8)
Supplement: Supplementary file 33 — Additional file 33. Verification of VviERF6L1 overexpression lines. (A) Semi-quant RT-qPCR of GFP, VviERF6L1, and VviUbi1 from leaves at cycle 32. (B) Verification of VviERF6L1 overexpression with RT-qPCR with VviGAPDH and VviACT7 reference genes from individual leaves represented as a normalized relative quantity, mean ± SE, n = 3 individual leaves from 3 individual plants. Stars indicate significance between G1 (empty vector control) and VviERF6L1 overexpression lines (p-value < 0.05) using student’s T-test. Blue and green corresponding to VviERF6L1 overexpression lines and empty vector control, respectively. [file 12864_2020_6811_MOESM33_ESM.pdf]

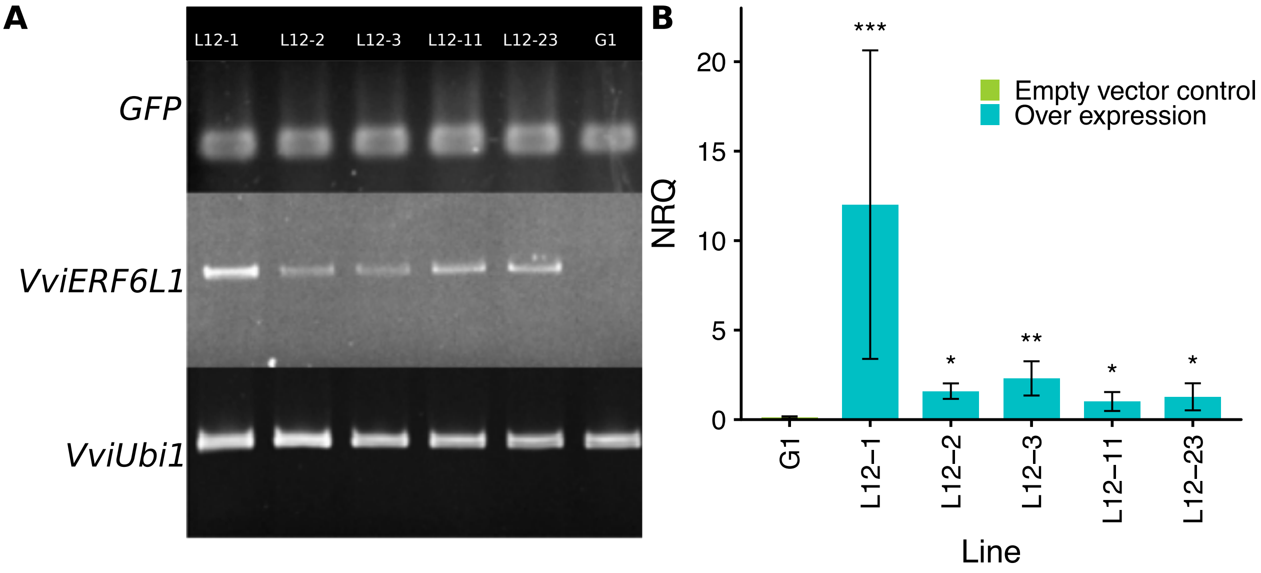

Additional File 33: Verification of *VviERF6L1* overexpression lines. (A) Semi-quant RT-qPCR of *GFP*, *VviERF6L1*, and *VviUbi1* from leaves at cycle 32. **(B)** Verification of *VviERF6L1* overexpression with RT-qPCR with *VviGAPDH* and *VviACT7* reference genes from individual leaves represented as a normalized relative quantity, mean ± SE, n = 3 individual leaves from 3 individual plants. Stars indicate significance between G1 (empty vector control) and *VviERF6L1* overexpression lines (p-value < 0.05) using student's T-test. Blue and green corresponding to *VviERF6L1* overexpression lines and empty vector control, respectively.
