# Supplementary material for: VviERF6Ls: an expanded clade in Vitis responds transcriptionally to abiotic and biotic stresses and berry development
Source: BMC Genomics. 2020 Jul 9;21:472. doi: 10.1186/s12864-020-06811-8 (PMC7350745; doi:10.1186/s12864-020-06811-8)
Supplement: Supplementary file 35 — Additional file 35. VviERF6L expression in VviERF6L1 overexpression lines. For each overexpression line (L12-3, L12-11, L12-23) and the empty vector control (G1), an average TMP value was calculated and log2 transformed and colored from yellow (low value) to purple (high value) for each of the 18 VviERF6Ls, n= 3. [file 12864_2020_6811_MOESM35_ESM.pdf]

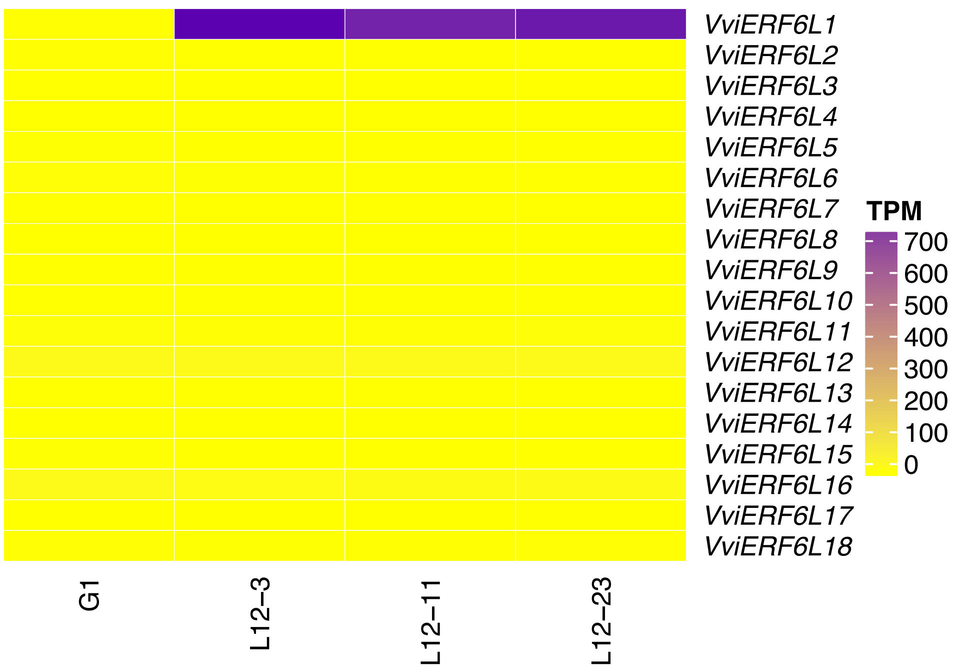

Additional File 35: *VviERF6L* expression in *VviERF6L1* overexpression lines. For each overexpression line (L12-3, L12-11, L12-23) and the empty vector control (G1), an average TMP value was calculated and $\log_2$ transformed and colored from yellow (low value) to purple (high value) for each of the 18 *VviERF6Ls*, n= 3.
