# Supplementary material for: VviERF6Ls: an expanded clade in Vitis responds transcriptionally to abiotic and biotic stresses and berry development
Source: BMC Genomics. 2020 Jul 9;21:472. doi: 10.1186/s12864-020-06811-8 (PMC7350745; doi:10.1186/s12864-020-06811-8)
Supplement: Supplementary file 37 — Additional file 37. Venn Diagram of differentially expressed genes between L12-3, -11, and -23 VviERF6L1 overexpression lines relative to G1 empty vector control. Number of upregulated genes presented in black and down regulated genes presented as grey. [file 12864_2020_6811_MOESM37_ESM.pdf]

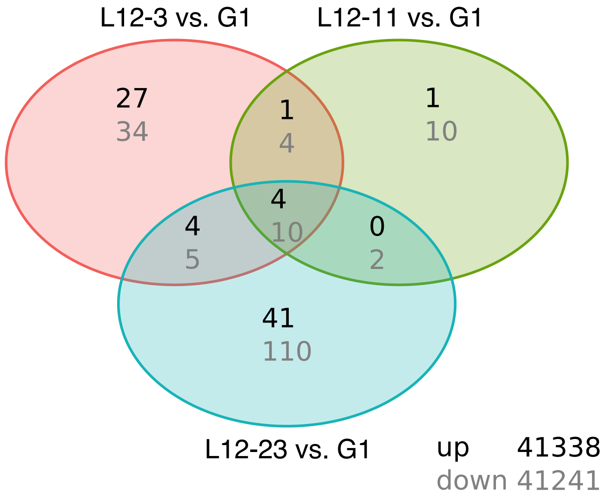

Additional File 37: Venn Diagram of differentially expressed genes between L12-3, -11, and -23 *VviERF6L1* overexpression lines relative to G1 empty vector control. Number of upregulated genes presented in black and down regulated genes presented as grey.
