# Supplementary material for: VviERF6Ls: an expanded clade in Vitis responds transcriptionally to abiotic and biotic stresses and berry development
Source: BMC Genomics. 2020 Jul 9;21:472. doi: 10.1186/s12864-020-06811-8 (PMC7350745; doi:10.1186/s12864-020-06811-8)
Supplement: Supplementary file 29 — Additional file 29. VviERF6L gene expression in Corvina pericarp over berry development across vineyards and years. Log2(RMA-normalized signal intensity+1) gene expression of 12 VviERF6Ls from Corvina pericarp from four representative Italian vineyards (abbreviated names from original paper; meaning nondisclosed) from 2006-2008 at veraison (V), mid-ripening (MR), and harvest (H) [GSE41633]; mean ± SE. [file 12864_2020_6811_MOESM29_ESM.pdf]

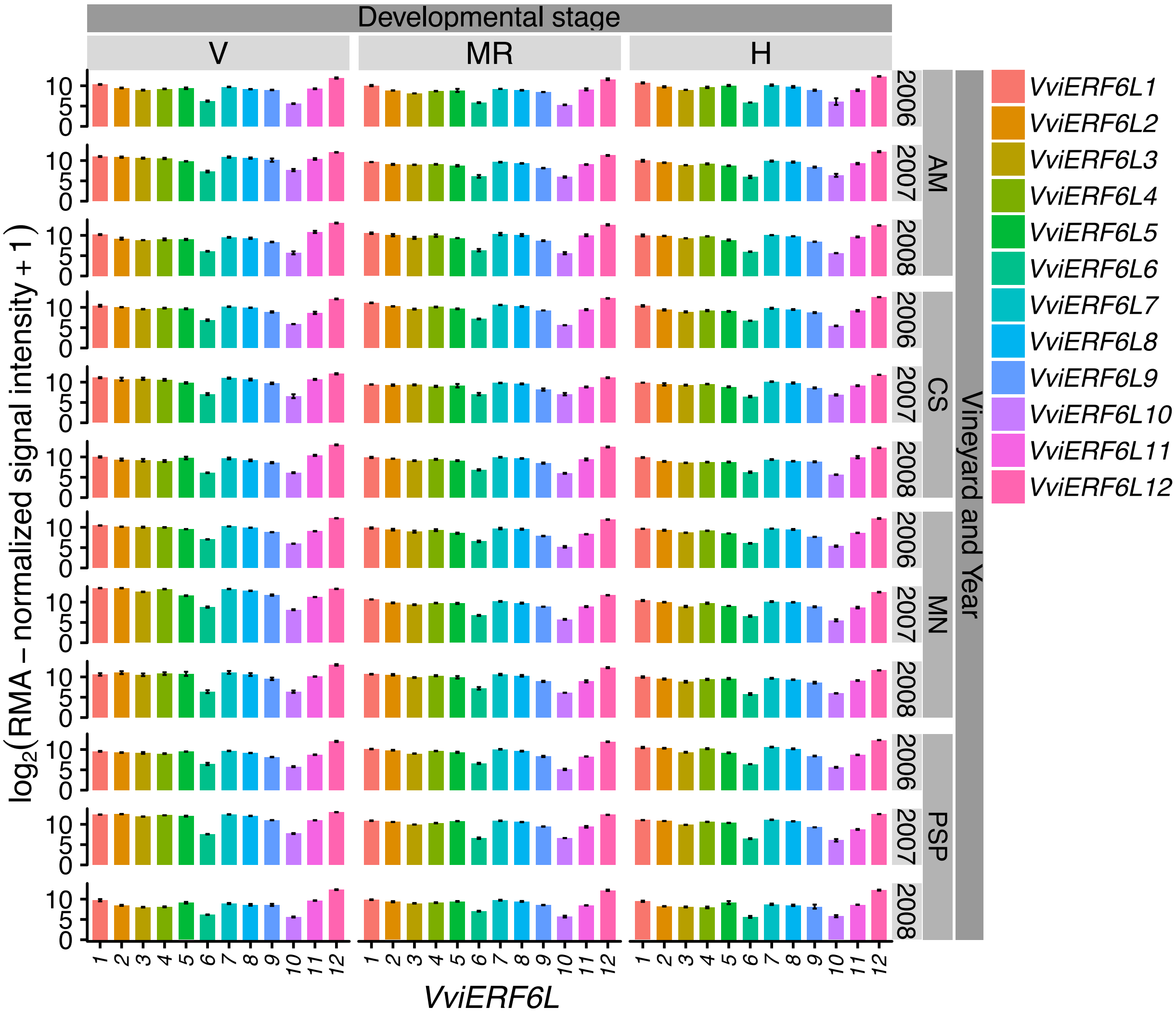

Additional File 29: *VviERF6L* gene expression in Corvina pericarp over berry development across vineyards and years. Log_2(RMA-normalized signal intensity+1) gene expression of 12 *VviERF6Ls* from Corvina pericarp from four representative Italian vineyards (abbreviated names from original paper; meaning nondisclosed) from 2006-2008 at veraison (V), mid-ripening (MR), and harvest (H) [GSE41633]; mean ± SE.
